# Facial masks affect emotion recognition in the general population and individuals with autistic traits

**DOI:** 10.1371/journal.pone.0257740

**Published:** 2021-09-30

**Authors:** Farid Pazhoohi, Leilani Forby, Alan Kingstone

**Affiliations:** Department of Psychology, University of British Columbia, Vancouver, British Columbia, Canada; University Hospitals Tubingen: Universitatsklinikum Tubingen, GERMANY

## Abstract

Facial expressions, and the ability to recognize these expressions, have evolved in humans to communicate information to one another. Face masks are equipment used in healthcare by health professionals to prevent the transmission of airborne infections. As part of the social distancing efforts related to COVID-19, wearing facial masks has been practiced globally. Such practice might influence affective information communication among humans. Previous research suggests that masks disrupt expression recognition of some emotions (e.g., fear, sadness or neutrality) and lower the confidence in their identification. To extend the previous research, in the current study we tested a larger and more diverse sample of individuals and also investigated the effect of masks on perceived intensity of expressions. Moreover, for the first time in the literature we examined these questions using individuals with autistic traits. Specifically, across three experiments using different populations (college students and general population), and the 10-item Autism Spectrum Quotient (AQ-10; lower and higher scorers), we tested the effect of facial masks on facial emotion recognition of anger, disgust, fear, happiness, sadness, and neutrality. Results showed that the ability to identify all facial expressions decreased when faces were masked, a finding observed across all three studies, contradicting previous research on fear, sad, and neutral expressions. Participants were also less confident in their judgements for all emotions, supporting previous research; and participants perceived emotions as less expressive in the mask condition compared to the unmasked condition, a finding novel to the literature. An additional novel finding was that participants with higher scores on the AQ-10 were less accurate and less confident overall in facial expression recognition, as well as perceiving expressions as less intense. Our findings reveal that wearing face masks decreases facial expression recognition, confidence in expression identification, as well as the perception of intensity for all expressions, affecting high-scoring AQ-10 individuals more than low-scoring individuals.

## Introduction

Charles Darwin’s 1872 book, *The Expression of Emotions in Man and Animals*, represents one of the first scientific attempts to study facial expressions. This work provided evidence that emotional expressions are adaptive and evolved to serve functions in the communication of information, including an individual’s affective state [1–4]. Since the revival of the importance of Darwin’s study of expressions of emotions during 1960s, there has been a surge of research on the perception and expression of emotions in primates, including humans [3].

Humans’ ability to recognize facial expressions is evident in infancy, and continues to develop during childhood and into adulthood [5, 6]. However, the affective information is not necessarily distributed homogeneously, with some regions of the face signalling more information regarding emotions than others [7, 8]. For example, individuals tend to inspect another’s mouth more when discerning happiness [9]. In general, humans use the mouth more than the eyes to both signal and discriminate facial expressions [9–11]. The recognition patterns of emotions from faces are generally similar among children, adults, and the elderly [12, 13].

Surgical masks (or simply face masks) are used by health professionals to prevent the transmission of airborne infections. As part of the social distancing efforts related to COVID-19, wearing facial masks has been practiced globally. For neurotypicals (NTs), the presence of facial masks results in decreased face recognition abilities when compared to non-masked faces [14–17], resulting in lower accuracy in emotion recognition in both adults [18–22] and children [23]. Moreover, masks cause more disruption than sunglasses in tasks requiring adults to recognize expressions and unfamiliar faces [21]. Facial masks also create confusion, in that disgust can be mistaken for anger, while happy, sad, and anger can be mistaken for neutral emotional expression [22]. For individuals with autism spectrum disorders (ASD), it could be anticipated that masks might create an even greater challenge where facial emotion recognition (FER) is concerned.

ASD are neurodevelopmental disorders that have the hallmark features of restricted interests, repetitive behaviours, and difficulties with social interactions [24]. Since Kanner [25] first described the cases of 11 autistic children and their inability to relate to, or connect with others, much research has been conducted on the perception of emotions in ASD. Disruptions in social interactions may be due, in part, to deficits in recognizing mental states in others [26, 27]. While the research is less clear for children and adolescents with autistic traits [28, 29], the majority of the extant research on adults with ASD suggests a general deficit in FER (for review see [30]). Specifically, when compared to NTs, individuals with autistic traits not only show reduced gaze duration and fixations on other people’s eyes [31–33; but see 34], they also spend more time looking at other people’s mouths [35, 36, but see 33], and rely more than NTs do on the mouth than the eyes for gauging emotions [30]. Additionally, they are more likely than NTs to begin their exploration of a face in the mouth region [33]. Researchers have also used the “Bubbles” method [37], or Gaussian holes, to occlude various regions of static faces displaying either fear or happiness. Using the “Bubble” method, Spezio et al. [38] found that when emotional information is reduced, high autistic trait individuals are more likely to look to the mouth to identify emotions. This reliance on the mouth region for categorizing emotions in others suggests that the increase in mask wearing for COVID-19 protection will have a greater negative impact on FER abilities for individuals with ASD than it will for the general public.

Although NTs [39], as well as those with autistic traits [40, 41], can use overall body language to facilitate emotion recognition [42, 43], we focus here on static images of the face. While previous studies have investigated the effect of facial mask on FER in NTs, to the best of our knowledge no research has tested facial emotion recognition as a function of facial mask in high autistic trait individuals. Therefore, in the current study we investigate the effect of facial masks on FER across three experiments using different populations (university students and the general population), and individual difference measures (low and high scorers on an autism quotient scale). Specifically, in Experiment 1 and using a large sample of university students, we test the effect of facial masks (or simply, masks) on FER, as well as the confidence in the recognition of the expressions and the perceived intensity level of those expressions. While recent studies have examined the effect of masks on FER [16, 18–22], only one [22] investigated participants’ confidence in their FER abilities with masked faces, and none of the studies asked participants to rate the intensity of emotions in masked conditions. In Experiments 2, we replicate the first experiment using a broader sample from the general population by recruiting participants from Mechanical Turk workers. Finally, in Experiment 3 we investigate the effects of facial masks as a function of high and low scores on the 10-item Autism Spectrum Quotient (AQ-10 [44]).

## Experiment 1

In the first experiment, we recruited university students to test the effect of masks and the sex of the stimuli on FER. We predict that facial masks will lead to a general decrease in FER across all emotions.

### Method

#### Participants

A total of 420 individuals (133 men and 287 women), aged between 18 and 36 years (M = 20.33, SD = 2.50), were recruited from the undergraduate Human Subject Pool at the University of British Columbia. Participants received course credit for participation. The study was approved by UBC ethics board.

#### Stimuli and procedure

**Fig 1:**
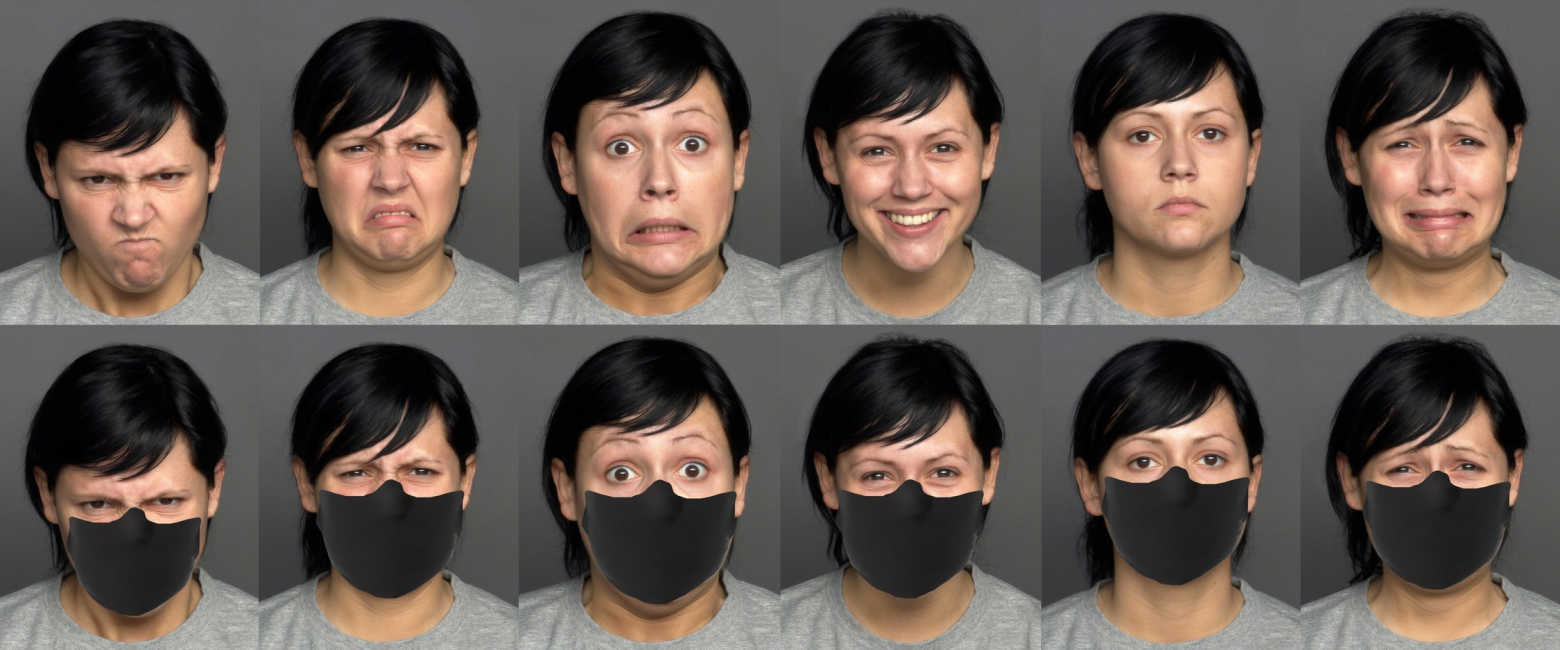
Example of six facial expressions of anger, disgust, fear, happiness, neutrality, and sadness (upper row) and their masked counterparts (lower row). Original material from top row stems from MPI FACES database (Ebner et al., 2010).

Images of eight male and eight female faces (aged between 19 and 31 years), each expressing anger, disgust, fear, happiness, sadness, or neutrality were obtained from the FACES database [45], resulting in 96 stimuli (16 faces x 6 expressions). Another set of 96 stimuli were created by superimposing a facial mask on the original images (see Fig 1 for an example). A within-subjects design was used, and participants randomly observed either the block with facial masks first or the block without the masks first.

After providing online consent to participate, participants were asked to identify the facial expressions in the images, given six choices: anger, disgust, fear, happy, neutral, and sad (“What is the facial expression of this person?”). Participants were also asked to use a sliding scale from 0 to 100 to indicate the level of confidence in their choice (“From 0 to 100%, how confident are you in your choice?”), as well as the perceived level of expressiveness or intensity of that expression (“From 0 to 100%, how much of this emotion is the person expressing?”). The question of expressiveness was not asked for faces that were judged to be neutral.

### Results

#### Facial expression recognition

A generalized linear mixed model was conducted to investigate the effects of facial mask and stimulus sex on the percentage of correct facial expression recognitions, with participant as a random factor. Due to the imbalance of males in our sample, we did not consider participant gender as an effect in our analysis. Results showed significant main effects for Mask and Stimulus Sex (Mask: *β* = 0.09, *SE* = 0.01, *χ²* (1) = 288.08, *z* = 16.97, *p* < .001; Stimulus Sex: *β* = -0.04, *SE* = 0.01, *χ²* (1) = 55.81, *z* = -7.47, *p* < .001). The main effects were qualified by a significant Mask × Stimulus Sex interaction (*β* = 0.05, *SE* = 0.01, *χ²* (1) = 24.07, *z* = 4.91, *p* < .001). Participants were significantly better in correct identification of unmasked male and female facial expressions (Male: *M* = 0.94, *SEM* = 0.01, 95% CI [.93, .95]; Female: *M* = 0.93, *SEM* = 0.01, 95% CI [.92, .94]) than masked male and female facial expressions, respectively (Male: *M* = 0.87, *SEM* = 0.01, 95% CI [.86, .89], *z* = -9.97, *p* < .001; Female: *M* = 0.81, *SEM* = 0.01, 95% CI [.79, .82], *z* = -13.87, *p* < .001); and better in recognition of masked male emotions than female masked emotions (*z* = 7.35, *p* < .001; see S1 Table in S1 File for the frequency and percentage of responses for each facial expression). No difference was observed for the correct identification between unmasked male and unmasked female facial expressions (*z* = 2.39, *p* = .101).

#### Confidence in facial expression recognition

A series of generalized linear mixed models were conducted to investigate the effect of facial mask on the confidence in FER. Each of the expressions were added as a fixed factor and participants as a random factor. Results showed significant main effects of Mask for confidence in all expressions (Anger: *β* = 0.12, *SE* = 0.01, *χ²* (1) = 3463.22, *z* = 58.85, *p* < .001; Disgust: *β* = 0.21, *SE* = 0.01, *χ²* (1) = 11435.80, *z* = 106.94, *p* < .001; Fear: *β* = 0.14, *SE* = 0.01, *χ²* (1) = 4746.90, *z* = 68.90, *p* < .001; Happiness: *β* = 0.22, *SE* = 0.01, *χ²* (1) = 12715.46, *z* = 112.76, *p* < .001; Neutral: *β* = 0.12, *SE* = 0.01, *χ²* (1) = 4010.09, *z* = 63.33, *p* < .001; Sadness: *β* = 0.12, *SE* = 0.01, *χ²* (1) = 5888.17, *z* = 76.73, *p* < .001). Participants were less confident in their recognition of facial expressions of disgust and neutrality behind the masks (Disgust: *M* = 66.15, *SEM* = 0.71, 95% CI [64.77, 67.57]; Neutral: *M* = 72.68, *SEM* = 0.93, 95% CI [70.87, 74.52];) compared to disgust and neutrality without masks (Disgust: *M* = 81.98, *SEM* = 0.88, 95% CI [80.27, 83.73]; Neutral: *M* = 82.21, *SEM* = 1.05, 95% CI [80.18, 84.30]).

**Fig 2:**
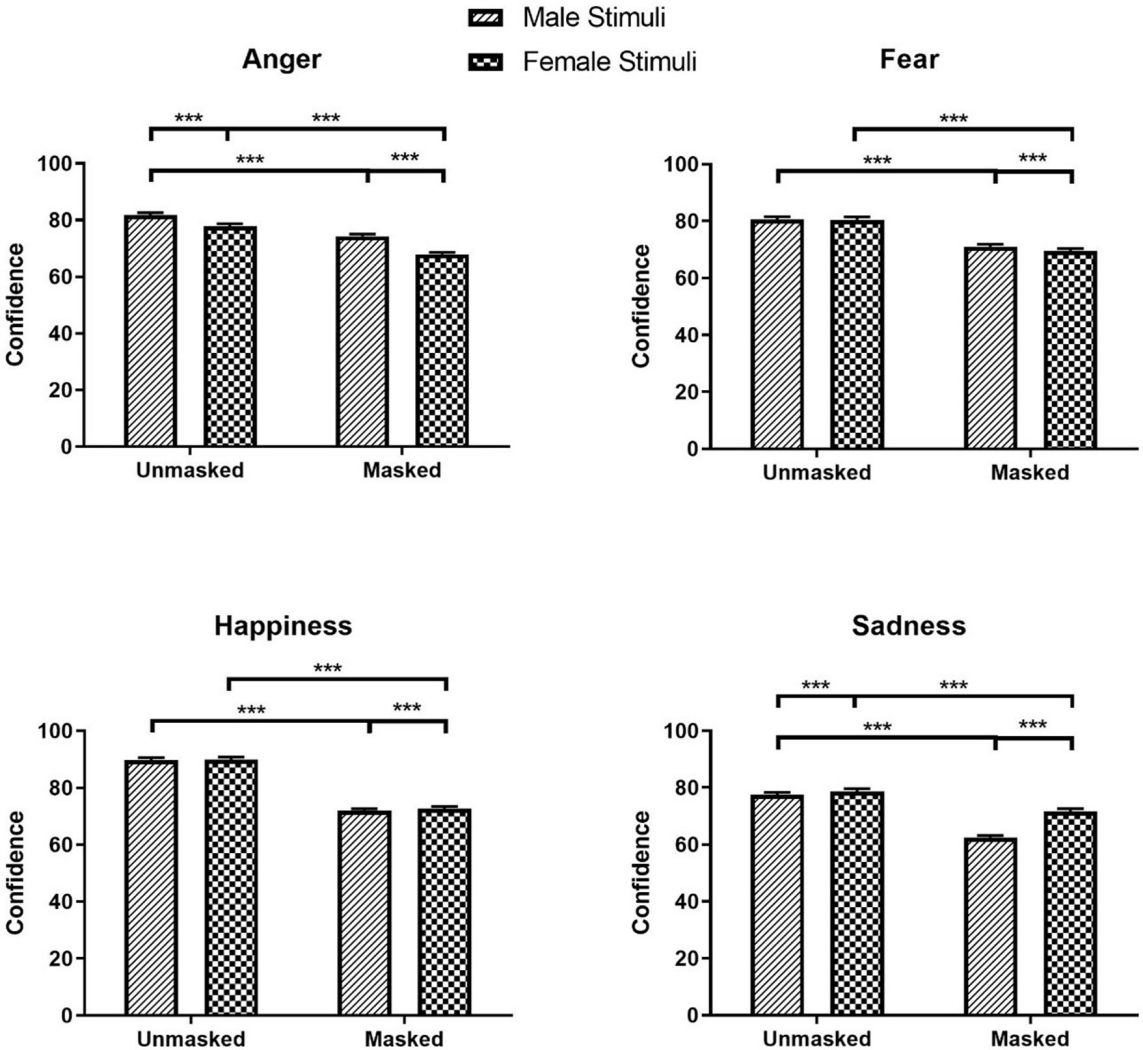
Confidence ratings for anger, fear, happiness, and sadness as a function of mask and stimuli sex. * *p* < 0.05, ** *p* < 0.01, *** *p* < 0.001.

Additionally, main effects of Stimulus Sex were significant for confidence in all expressions (Anger: *β* = -0.07, *SE* = 0.01, *χ²* (1) = 1211.80, *z* = -34.81, *p* < .001; Disgust: *β* = -0.04, *χ²* = 422.00, *z* = -20.54, *p* < .001; Fear: *β* = -0.01, *SE* = 0.01, *χ²* (1) = 32.61, *z* = -5.71, *p* < .001; Happiness: *β* = 0.01, *SE* = 0.01, *χ²* (1) = 12.73, *z* = 3.57, *p* < .001; Neutral: *β* = -0.04, *SE* = 0.01, *χ²* (1) = 371.43, *z* = -19.27, *p* < .001; Sadness: *β* = 0.08, *SE* = 0.01, *χ²* (1) = 1455.09, *z* = 38.15, *p* < .001). Participants were more confident in recognition of disgust and neutrality in male faces (Disgust: *M* = 75.18, *SEM* = 0.81, 95% CI [73.61, 76.78]; Neutral: *M* = 78.76, *SEM* = 1.01, 95% CI [76.81, 80.76]) than female faces (Disgust: *M* = 72.14, *SEM* = 0.78, 95% CI [70.63, 73.69]; Neutral: *M* = 75.86, *SEM* = 0.97, 95% CI [73.98, 77.79]). The significant main effects of Mask and Stimulus Sex for anger, fear, happiness, and sadness were qualified by significant Mask × Stimulus Sex interactions for confidence in anger, fear, happiness, and sadness (Anger: *β* = 0.04, *SE* = 0.01, *χ²* (1) = 109.89, *z* = 10.48, *p* < .001; Fear: *β* = 0.02, *SE* = 0.01, *χ²* (1) = 25.94, *z* = 5.09, *p* < .001; Happiness: *β* = -0.01, *SE* = 0.01, *χ²* (1) = 6.33, *z* = -2.52, *p* = .012; Sadness: *β* = -0.12, *SE* = 0.01, *χ²* (1) = 920.42, *z* = -30.34, *p* < .001). Participants were more confident in identifying anger and fear in masked male faces (Anger: *M* = 75.25, *SEM* = 0.83, 95% CI [72.64, 75.90]; Fear: *M* = 71.00, *SEM* = 0.88, 95% CI [69.29, 72.75]) than masked female faces (Anger: *M* = 67.88, *SEM* = 0.77, 95% CI [66.39, 69.39], *z* = 30.97, *p* < .001; Fear: *M* = 69.50, *SEM* = 0.87, 95% CI [67.82, 71.22], *z* = 7.36, *p* < .001), while they were more confident in identifying happiness and sadness in masked female faces (Happiness: *M* = 72.71, *SEM* = 0.77, 95% CI [71.21, 74.24]; Sadness: *M* = 71.70, *SEM* = 0.85, 95% CI [70.06, 73.38]) than masked male faces (Happiness: *M* = 71.87, *SEM* = 0.76, 95% CI [70.39, 73.37], *z* = -4.07, *p* < .001; Sadness: *M* = 62.47, *SEM* = 0.74, 95% CI [61.04, 63.92], *z* = -46.56, *p* < .001; see Fig 2). No significant difference was observed for confidence in fear and happiness between unmasked male and female faces (Fear: *z* = 0.45; Happiness: *z* = -0.79; *p*s = .999).

#### Intensity of expression

To investigate the effect of facial masks on the intensity of facial expressions, a series of generalized linear mixed models were conducted with each of the expressions as a fixed factor and participants as a random factor. Note that the question of expression intensity was not included for expressions judged to be neutral. Results showed significant main effects of Mask for intensity in all expressions (Anger: *β* = 0.06, *SE* = 0.01, *χ²* (1) = 895.82, *z* = 29.93, *p* < .001; Disgust: *β* = 0.16, *SE* = 0.01, *χ²* (1) = 6189.82, *z* = 78.68, *p* < .001; Fear: *β* = 0.07, *SE* = 0.01, *χ²* (1) = 1264.25, *z* = 35.56, *p* < .001; Happiness: *β* = 0.27, *SE* = 0.01, *χ²* (1) = 16730.60, *z* = 129.40, *p* < .001; Sadness: *β* = 0.12, *SE* = 0.01, *χ²* (1) = 3142.15, *z* = 56.05, *p* < .001); participants perceived anger and disgust expressions behind the masks (Anger: *M* = 66.49, *SEM* = 0.78, 95% CI [64.98, 68.03]; Disgust: *M* = 66.45, *SEM* = 0.70, 95% CI [65.10, 67.83];) as less expressive (lower intensity) than those without masks (Anger: *M* = 70.73, *SEM* = 0.83, 95% CI [69.13, 72.38]; Disgust: *M* = 77.94, *SEM* = 0.82, 95% CI [76.36, 79.56].

Moreover, the main effects of Stimulus Sex were significant for intensity of all expressions (Anger: *β* = -0.07, *SE* = 0.01, *χ²* (1) = 1114.31, *z* = -33.38, *p* < .001; Disgust: *β* = -0.05, *SE* = 0.01, *χ²* (1) = 680.47, *z* = -26.09, *p* < .001; Fear: *β* = -0.04, *SE* = 0.01, *χ²* (1) = 445.53, *z* = -21.11, *p* < .001; Happiness: *β* = -0.02, *SE* = 0.01, *χ²* (1) = 141.20, *z* = -11.88, *p* < .001; Sadness: *β* = 0.04, *SE* = 0.01, *χ²* (1) = 418.12, *z* = 20.45, *p* < .001). Participants perceived male faces (Anger: *M* = 70.99, *SEM* = 0.83, 95% CI [69.38, 72.63]; Disgust: *M* = 73.90, *SEM* = 0.77, 95% CI [72.40, 75.43]) as more expressive than female faces in anger and disgust (Anger: *M* = 66.25, *SEM* = 0.78, 95% CI [64.78, 67.79]; Disgust: *M* = 70.09, *SEM* = 0.73, 95% CI [68.66, 71.54]).

**Fig 3:**
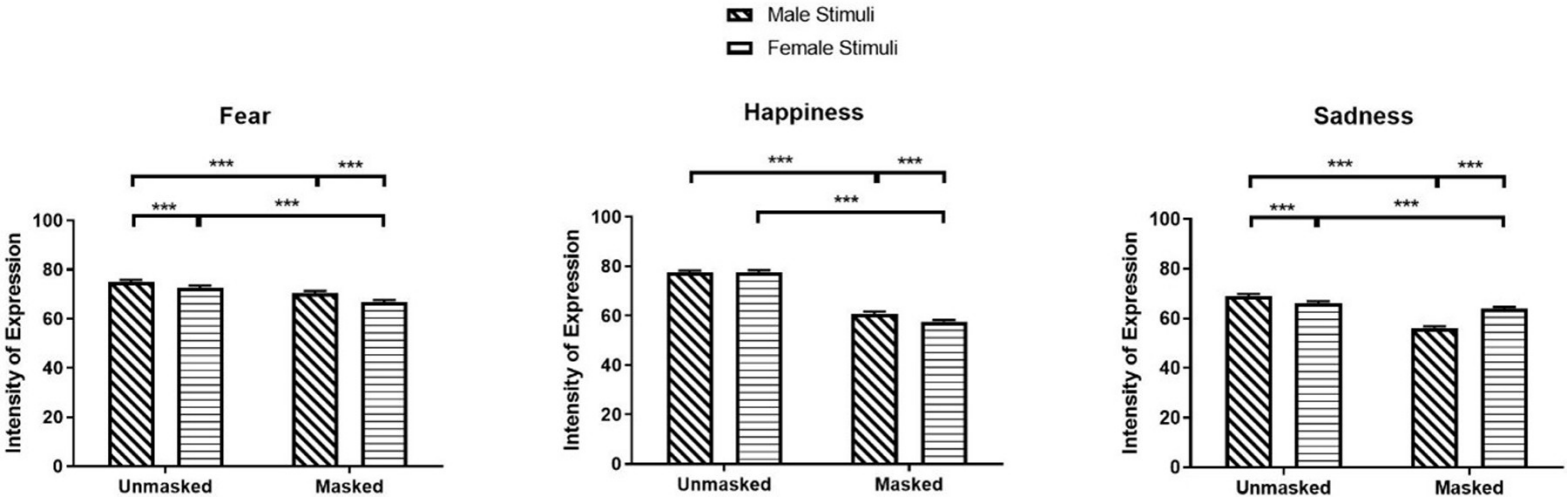
Intensity of expression ratings for fear, happiness, and sadness as a function of mask and stimuli sex. * *p* < 0.05, ** *p* < 0.01, *** *p* < 0.001.

The significant main effects of Mask and Stimulus Sex for fear, happiness, and sadness were qualified by significant Mask × Stimulus Sex interactions for intensity of expression in fear, happiness, and sadness (Fear: *β* = 0.02, *SE* = 0.01, *χ²* (1) = 32.39, *z* = 5.69, *p* < .001; Happiness: *β* = 0.06, *SE* = 0.01, *χ²* (1) = 204.14, *z* = 14.29, *p* < .001; Sadness: *β* = -0.17, *SE* = 0.01, *χ²* (1) = 1615.27, *z* = -40.19, *p* < .001). Participants rated fear and happiness in masked male faces (Fear: *M* = 70.55, *SEM* = 0.85, 95% CI [68.91, 72.23]; Happiness: *M* = 60.83, *SEM* = 0.76, 95% CI [59.36, 62.34]) as more expressive than masked female faces (Fear: *M* = 66.81, *SEM* = 0.80, 95% CI [65.26, 68.41], *z* = 18.53, *p* < .001; Happiness: *M* = 57.60, *SEM* = 0.72, 95% CI [56.20, 59.03], *z* = 17.30, *p* < .001), while they rated sadness in masked female faces (*M* = 63.92, *SEM* = 0.82, 95% CI [62.33, 65.55]) as more expressive compare to masked male faces (*M* = 56.15, *SEM* = 0.72, 95% CI [54.76, 57.58], *z* = -41.56, *p* < .001; see Fig 3). No significant difference was observed for intensity in happiness between unmasked male and female faces (*z* = -1.84, *p* = .396).

### Discussion

Using a sample of university students, the effect of facial masks on FER was investigated and the results showed that participants were better in FER of unmasked faces than masked faces. Furthermore, participants were better in FER in male masked faces than in female masked faces. Participants were less confident in their recognition of all investigated expressions behind the masks than facial expressions without masks. The overall confidence in FER was also sensitive to the sex of the faces, as participants were more confident in recognition of anger, disgust, fear, and neutrality in male faces, but more confident in recognition of happiness and sadness in female faces. When faces were masked, participants were more confident in their identification of anger and fear in male faces than in female faces, while they were more confident in identifying sadness in masked female faces than in masked male faces. Yet, all of the emotions were rated as less expressive when under a mask. While male faces were rated as more expressive in anger, disgust, fear, and happiness, and female faces were more expressive in sadness, participants rated fear and happiness in masked male faces as more expressive than in masked female faces, and rated sadness in masked female faces as more expressive than in masked male faces.

Using a database similar to the one used in the current study, Carbon [22] did not find a significant difference between masked and unmasked fear and neutral expressions. One potential explanation for this difference between our study and Carbon’s concerns the number of stimuli used and participants tested. While in the Carbon study 41 individuals rated the emotions of 12 identities (a combination of young, middle-aged, and older faces), we recruited 420 participants to identify the emotions of 16 young identities. Our larger population, as well as our larger set of stimuli within a coherent age group, may have yielded a more robust effect, revealing that recognition of all emotions, including fear and neutral faces, are affected by wearing a mask. Moreover, by using a larger sample size, our analyses had the power to test for the interactions between mask and stimulus sex on FER. However, there are still limitations to our study, for example, using a sample of only university students. Therefore, in the next study we aimed to test the effect of facial masks on FER within a more general population.

## Experiment 2

Experiment 2 tested if the results of Experiment 1 generalise to a broader population. We recruited participants from Amazon Mechanical Turk workers.

### Method

#### Participants

A total of 199 individuals (130 men and 69 women), aged between 18 and 73 years (*M* = 34.58, *SD* = 10.18) were recruited through Amazon Mechanical Turk (MTurk) from the United States to complete an online survey. Participants provided online consent to participate. MTurk’s robustness is extensively researched and results support it as a valid means of collecting online data for the behavioural sciences, sometimes even superior to in-person collection [46]. MTurk subjects are often more representative of the U.S. population than in-person convenience samples, but they might be less representative than subjects in Internet panels or national probability sampling [46–48]. A total of 141 participants (70.9%) reported being married, and 3.5% as being divorced, widowed, or separated; an additional 19.6% reported being single, and 6.0% in a relationship. In terms of their highest academic degree, 10.1% had a high school diploma, 6.0% had a post-secondary diploma, 47.7% of the participants had an undergraduate degree, and 35.7% had a post-graduate degree (MA or PhD).

#### Stimuli and procedure

Twelve young faces, six male and six female, with six emotional expressions (anger, disgust, fear, happiness, sadness, and neutral) were obtained from the FACES database [45], resulting in 72 stimuli. Another set of 72 stimuli were created by superimposing a facial mask on the original photos. The faces used in this experiment were a subset of faces used in Experiment 1. The reduction of stimuli (a total of 192 trials in Experiment 1 to 144 in Experiment 2) was to reduce the length of the study and the risk of participants dropping out [49, 50]. A within-subjects design was used, and participants randomly observed either the block with facial masks first or the block without the masks first. The rest of the procedure, including the questions asked, was as in Experiment 1.

### Results

#### Facial expression recognition

A generalized linear mixed model was conducted to investigate the effect of facial masks, and stimulus sex on the percentage of correct facial expression recognitions, with participant as a random factor. Once again, due to the imbalance of males in our sample, we did not consider participant gender as an effect in our analysis. Results showed significant main effects for Mask and Stimulus Sex (Mask: *β* = 0.14, *SE* = 0.01, *χ²* (1) = 165.76, *z* = 12.87, *p* < .001; Stimulus Sex: *β* = 0.05, *SE* = 0.01, *χ²* (1) = 18.64, *z* = 4.32, *p* < .001). These main effects were qualified by a significant Mask × Stimulus Sex interaction (*β* = -0.06, *SE* = 0.02, *χ²* (1) = 8.83, *z* = -2.97, *p* = .003). Participants were significantly poorer in correct identification of expressions in masked female faces (*M* = 0.59, *SEM* = 0.02, 95% CI [.55, .63];) than expressions in masked male faces (*M* = 0.67, *SEM* = 0.02, 95% CI [.63, .71], *z* = -4.36, *p* < .001) and unmasked female faces (*M* = 0.77, *SEM* = 0.02, 95% CI [.73, .80], *z* = -10.99, *p* < .001); and poorer in recognition of masked male emotions than unmasked male emotions (*M* = 0.78, *SEM* = 0.02, 95% CI [.74, .82], *z* = -7.59, *p* < .001; see S2 Table in S1 File for the frequency and percentage of responses for each facial expression). No difference was observed for the correct identification between unmasked male and unmasked female facial expressions (*z* = -1.26, *p* = .999).

#### Confidence in facial expression recognition

A series of generalized linear mixed models were conducted to investigate the effect of masks on the confidence in facial expression recognition. Each of the expressions were added as a fixed factor and participants as a random factor. Similar to the results of Experiment 1, participants in Experiment 2 were less confident in their recognition of the facial expressions behind the masks in all expressions (Anger: *M* = 74.18, *SEM* = 1.00, 95% CI [72.25, 76.16]; Disgust: *M* = 71.84, *SEM* = 0.98, 95% CI [69.94, 73.79]; Fear: *M* = 75.98, *SEM* = 1.07, 95% CI [73.92, 78.10]; Happiness: *M* = 73.57, *SEM* = 1.04, 95% CI [71.57, 75.63]; Neutral: *M* = 72.52, *SEM* = 1.11, 95% CI [70.38, 74.73]; Sadness: *M* = 71.56, *SEM* = 1.01, 95% CI [69.62, 73.56]) compared to the facial expressions without masks (Anger: *M* = 76.96, *SEM* = 1.03, 95% CI [74.96, 79.02], *β* = 0.04, *SE* = 0.01, *χ²* (1) = 124.39, *z* = 11.15, *p* < .001; Disgust: *M* = 78.50, *SEM* = 1.07, 95% CI [76.43, 80.63], *β* = 0.09, *SE* = 0.01, *χ²* (1) = 716.86, *z* = 26.77, *p* < .001; Fear: *M* = 78.33, *SEM* = 1.10, 95% CI [76.20, 80.52], *β* = 0.03, *SE* = 0.01, *χ²* (1) = 86.89, *z* = 9.32, *p* < .001; Happiness: *M* = 82.33, *SEM* = 1.16, 95% CI [80.09, 84.63], *β* = 0.11, *SE* = 0.01, *χ²* (1) = 1194.26, *z* = 34.56, *p* < .001; Neutral: *M* = 77.23, *SEM* = 1.18, 95% CI [74.95, 79.58], *β* = 0.06, *SE* = 0.01, *χ²* (1) = 360.72, *z* = 18.99, *p* < .001; Sadness: *M* = 76.42, *SEM* = 1.07, 95% CI [74.34, 78.55], *β* = 0.07, *SE* = 0.01, *χ²* (1) = 387.38, *z* = 19.68, *p* < .001). However, participants were more confident in recognition of disgust and neutrality in male faces (Disgust: *M* = 75.54, *SEM* = 1.03, 95% CI [73.54, 77.59]; Neutral: *M* = 75.16, *SEM* = 1.15, 95% CI [72.94, 77.45]) than in female faces (Disgust: *M* = 74.66, *SEM* = 1.02, 95% CI [72.69, 76.69], *β* = 0.01, *χ²* = 12.39, *z* = 3.52, *p* < .001; Neutral: *M* = 74.51, *SEM* = 1.14, 95% CI [72.31, 76.78], *β* = 0.01, *χ²* = 6.90, *z* = 2.63, *p* = .009)

**Fig 4:**
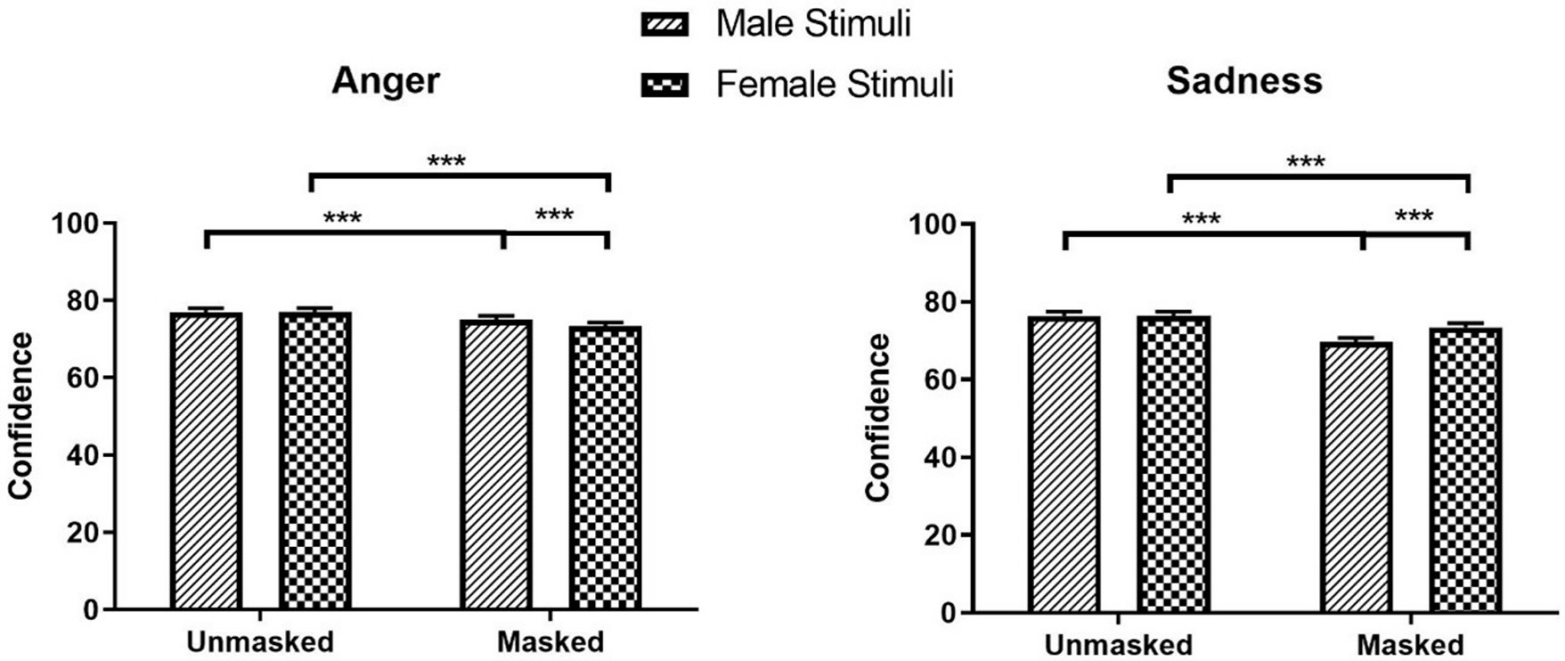
Confidence ratings for anger and sadness as a function of mask and stimuli sex. * *p* < 0.05, ** *p* < 0.01, *** *p* < 0.001.

The significant main effects of Mask and Stimulus Sex for anger and sadness were qualified by significant Mask × Stimulus Sex interactions for confidence in anger and sadness (Anger: *β* = -0.02, *SE* = 0.01, *χ²* (1) = 13.30, *z* = -3.65, *p* < .001; Sadness: *β* = 0.05, *SE* = 0.01, *χ²* (1) = 60.99, *z* = 7.81, *p* < .001). Participants were more confident in identifying anger in masked male faces (*M* = 75.04, *SEM* = 1.02, 95% CI [73.06, 77.07]) than in masked female faces (*M* = 73.33, *SEM* = 1.00, 95% CI [71.39, 75.32], *z* = -4.89, *p* < .001), while they were more confident in identifying sadness in masked female faces (*M* = 73.45, *SEM* = 1.05, 95% CI [71.43, 75.53]) than in masked male faces (*M* = 69.71, *SEM* = 0.99, 95% CI [67.79, 71.69], *z* = 10.90, *p* < .001; see Fig 4). No significant difference was observed for confidence in anger and sadness between unmasked male and female faces (Anger: *z* = 0.22; Sadness: *z* = 0.03; *p*s = .999).

#### Intensity of expression

To investigate the effect of facial masks on the perception of intensity of facial expressions, a series of generalized linear mixed models were conducted with each of the expressions as a fixed factor and participants as a random factor. Results showed significant main effects of Mask for intensity in all expressions (Anger: *β* = 0.04, *SE* = 0.01, *χ²* (1) = 115.12, *z* = 282.38, *p* < .001; Disgust: *β* = 0.08, *SE* = 0.01, *χ²* (1) = 547.82, *z* = 23.41, *p* < .001; Fear: *β* = 0.02, *SE* = 0.01, *χ²* (1) = 27.23, *z* = 5.22, *p* < .001; Happiness: *β* = 0.14, *SE* = 0.01, *χ²* (1) = 1818.54, *z* = 42.64, *p* < .001; Sadness: *β* = 0.06, *SE* = 0.01, *χ²* (1) = 300.90, *z* = 17.35, *p* < .001); participants perceived facial expressions of disgust and fear under the masks (Disgust: *M* = 69.14, *SEM* = 0.98, 95% CI [67.25, 71.09]; Fear: *M* = 72.41, *SEM* = 1.07, 95% CI [70.34, 74.54]) as less expressive (less intense) than those without masks (Disgust: *M* = 74.84, *SEM* = 1.06, 95% CI [72.79, 76.94]; Fear: *M* = 73.69, *SEM* = 1.09, 95% CI [71.58, 75.86]). Moreover, the main effects of Stimulus Sex were significant for intensity of anger, disgust, and happiness (Anger: *β* = 0.01, *χ²* = 11.71, *z* = 3.42, *p* < .001; Disgust: *β* = 0.08, *χ²* = 25.59, *z* = 5.06, *p* < .001; Happiness: *β* = 0.02, *χ²* = 33.93, *z* = 5.83, *p* < .001); participants perceived male faces (Disgust: *M* = 72.55, *SEM* = 1.03, 95% CI [70.56, 74.59]) as more expressive than female faces in disgust (Disgust: *M* = 71.32, *SEM* = 1.01, 95% CI [69.37, 73.33]).

**Fig 5:**
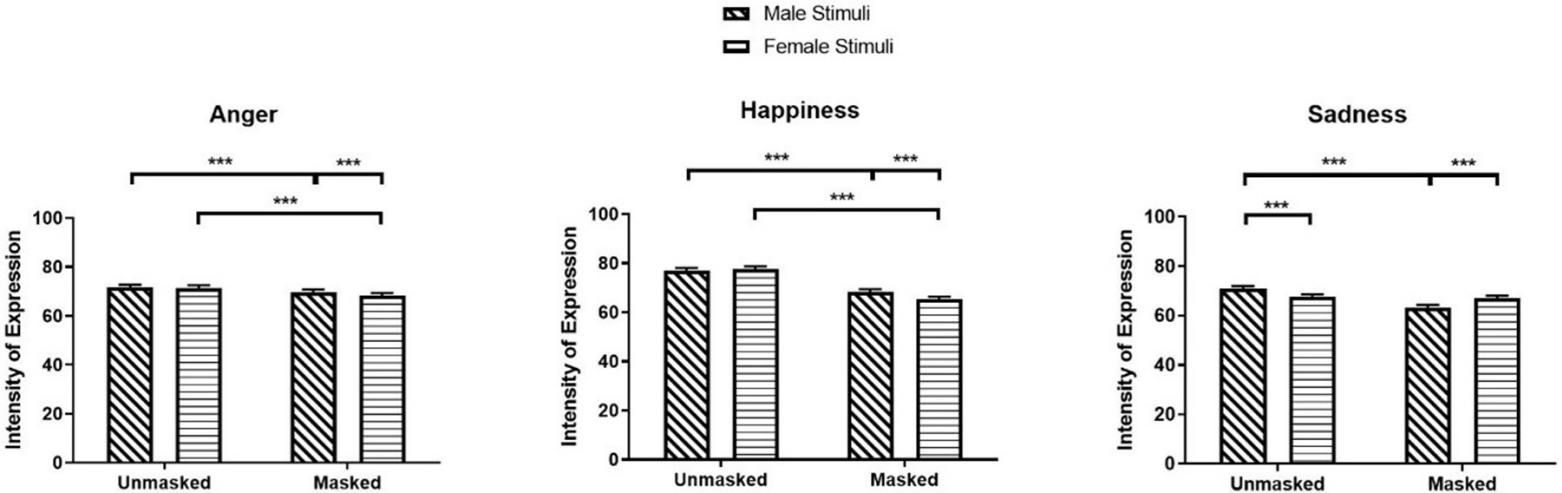
Intensity of expression ratings for anger, happiness, and sadness as a function of mask and stimuli sex. * *p* < 0.05, ** *p* < 0.01, *** *p* < 0.001.

The significant main effects of Mask and Stimulus Sex for anger, happiness, and sadness were qualified by significant Mask × Stimulus Sex interactions for intensity in happiness and sadness (Anger: *β* = -0.02, *SE* = 0.01, *χ²* (1) = 5.58, *z* = -2.36, *p* = .018; Happiness: *β* = -0.05, *SE* = 0.01, *χ²* (1) = 62.68, *z* = -7.92, *p* < .001; Sadness: *β* = 0.10, *SE* = 0.01, *χ²* (1) = 221.20, *z* = 14.87, *p* < .001). Participants rated anger and happiness in masked male faces (Anger: *M* = 69.68, *SEM* = 1.07, 95% CI [67.61, 71.80]; Happiness: *M* = 68.48, *SEM* = 1.01, 95% CI [67.75, 71.82]) as more expressive than in masked female faces (Anger: *M* = 68.31, *SEM* = 1.05, 95% CI [66.29, 70.40]; Happiness: *M* = 65.37, *SEM* = 0.97, 95% CI [63.50, 67.29], *z* = -9.38, *p* < .001), while they rated sadness in masked female faces (*M* = 66.93, *SEM* = 1.09, 95% CI [64.83, 69.11]) as more intense than masked male faces (*M* = 63.27, *SEM* = 1.15, 95% CI [61.28, 65.33], *z* = 11.21, *p* < .001; see Fig 5). No significant difference was observed for intensity in anger and happiness between unmasked male and female faces (Anger: *z* = -0.76; Happiness: *z* = 1.54; *p*s > .747), or between unmasked and masked sad female faces (*z* = -1.75; *p* = .479).

### Discussion

The second experiment aimed to test if the results of Experiment 1, which used a university student sample, replicate in the general population. Results from Mechanical Turk workers showed that similar to Experiment 1, participants’ FER decreased significantly as a function of facial mask, and that participants were less confident in their recognition of the facial expressions behind the masks. This significant difference was observed for all expressions. Moreover, all facial expressions under the masks were rated as less expressive than those without masks, and male faces were considered more expressive in anger, disgust, and happiness compared to female faces.

## Experiment 3

Our final study examined the effect of a facial mask on FER for high autistic trait individuals. Given that individuals with autistic traits place more importance than NTs do on the mouth to recognize facial expressions, we predict that a facial mask will make FER particularly difficult for people with autistic traits. Specifically, they will be less accurate than NTs at FER, and that masks will negatively impact their FER accuracy more than they do NTs’.

### Method

#### Participants

One hundred and forty-two participants were recruited from the Human Subject Pool at the University of British Columbia according to the school’s ethical guidelines, and received extra credit for agreeing to participate. Participants provided online consent to participate. All participants completed the 10-item Autism Spectrum Quotient (AQ-10; [44]) prior to the experiment, and they had no way of knowing that their AQ-10 score was relevant to the present study. The AQ-10 includes 10 items from the 50-item Autism Spectrum Quotient (AQ; [26]), and was designed to be a quick and effective screening method for ASD. The AQ-10 scores reliably in specificity (0.91), sensitivity (0.88), and has a positive predictive value of 0.85 [44]. An individual who scores 6 or higher on the AQ-10 is referred for a full diagnostic assessment. For individuals with ASD, the AQ-10 yields a mean score of 7.93, and for NTs a mean score of 2.77 [44]. Thus, higher scores on the AQ-10 indicate more autistic traits have been endorsed.

Participants were divided into two groups: “high scorers”, comprised of participants who scored 6 or higher on the AQ-10, and “low scorers”, which included participants who scored 5 or lower on the AQ-10. This division yielded 71 participants (50 women) between the ages of 18 and 29 (*M* = 20.39, *SD* = 2.27) in the high scorers’ group, and 71 (51 women) between the ages of 18 and 39 (*M* = 20.52, *SD* = 2.98) in the low scorers’ group.

#### Stimuli and procedure

The same stimuli and procedures were used from Experiment 1.

### Results

#### Facial expression recognition

A linear generalized mixed model was conducted to investigate the effects of AQ-10 (high vs. low score) and masks on the percentage of correct facial expression recognitions, with participants as a random factor. As in the previous two experiments, we did not consider participant gender as an effect in our analysis due to the imbalance of males in our sample. Results showed significant main effects for Mask and AQ-10, however their interaction was not significant (Mask: *β* = 0.09, *SE* = 0.01, *χ²* (1) = 99.05, *z* = 9.95, *p* < .001; AQ-10: *β* = -0.05, *SE* = 0.01, *χ²* (1) = 31.84, *z* = -5.64, *p* < .001; Mask × AQ-10: *β* = -0.01, *SE* = 0.02, *χ²* (1) = 0.04, *z* = -0.21, *p* = .837). Participants were significantly better in facial expression recognition (FER) of unmasked facial expressions (*M* = 0.95, *SEM* = 0.01, 95% CI [.94, .96]) than masked facial expressions (*M* = 0.86, *SEM* = 0.01, 95% CI [.85, .88]). Moreover, results showed those participants who scored 5 or lower (“low scorers”) on the AQ-10 (*M* = 0.93, *SEM* = 0.01, 95% CI [.92, .94]) were significantly better in FER compared to high scorers (those who scored 6 or above) on the AQ-10 (*M* = 0.88, *SEM* = 0.01, 95% CI [.87, .89]).

The main effects of AQ10 and Stimulus Sex were qualified by a significant AQ10 × Sex Stimulus interaction, *β* = -0.05, *SE* = 0.02, *χ²* (1) = 8.72, *z* = -2.95, *p* = .003: High scorers were better at FER for male stimuli (*M* = 0.91, *SEM* = 0.01, 95% CI [.90, .93]) than in female stimuli (*M* = 0.85, *SEM* = 0.01, 95% CI [.83, .87], *z* = 4.62, *p* < .001) Additionally, high scorers were significantly poorer in recognition of female FER compared to low scorers (*M* = 0.92, *SEM* = 0.01, 95% CI [.91, .94], *z* = 5.59, *p* < .001). No other interaction was observed. See S3 Table in S1 File for the frequency and percentage of responses for each facial expression as a function of facial mask, AQ-10, and stimulus sex. No difference was observed for the correct expression identification of male and female faces for low scorers (z = -0.99; *p* = .999), and between low and high scorers for male faces (*z* = 2.05; *p* = .240).

#### Confidence in facial expression recognition

A series of generalized linear mixed models were conducted to investigate the effect of masks on the confidence in facial expression recognition, as a function of AQ-10 and Stimulus sex. Each of the expressions were added as a fixed factor and participants as a random factor. Result showed significant main effects of Mask for anger, disgust, fear happiness, neutrality, and sadness (Anger: *β* = 0.10, *SE* = 0.01, *χ²* (1) = 784.25, *z* = 28.00, *p* < .001; Disgust: *β* = 0.20, *SE* = 0.01, *χ²* (1) = 3343.21, *z* = 57.82, *p* < .001; Fear: *β* = 0.13, *SE* = 0.01, *χ²* (1) = 1380.68, *z* = 37.16, *p* < .001; Happiness: *β* = 0.21, *SE* = 0.01, *χ²* (1) = 3934.59, *z* = 62.73, *p* < .001; Neutral: *β* = 0.11, *SE* = 0.01, *χ²* (1) = 1180.21, *z* = 34.35, *p* < .001; Sadness: *β* = 0.16, *SE* = 0.01, *χ²* (1) = 2086.57, *z* = 45.68, *p* < .001), while the main effect of AQ-10 for anger, disgust, happiness, and neutrality were significant (Anger: *β* = -0.09, *SE* = 0.04, *χ²* (1) = 6.03, *z* = -2.46, *p* = .014; Disgust: *β* = -0.11, *SE* = 0.04, *χ²* (1) = 8.38, *z* = -2.89, *p* < .001; Happiness: *β* = -0.10, *SE* = 0.03, *χ²* (1) = 10.77, *z* = -3.28, *p* = .001; Neutral: *β* = -0.10, *SE* = 0.01, *χ²* (1) = 6.78, *z* = -2.60, *p* = .009). The effects of Stimulus Sex for anger, disgust, neutrality, and sadness were significant (Anger: *β* = -0.07, *SE* = 0.01, *χ²* (1) = 373.98, *z* = -19.34, *p* < .001; Disgust: *β* = -0.03, *SE* = 0.01, *χ²* (1) = 75.19, *z* = -8.67, *p* < .001; Neutral: *β* = -0.05, *SE* = 0.01, *χ²* (1) = 228.38, *z* = -15.11, *p* < .001; Sadness: *β* = 0.09, *SE* = 0.01, *χ²* (1) = 674.16, *z* = 25.96, *p* < .001).

**Fig 6:**
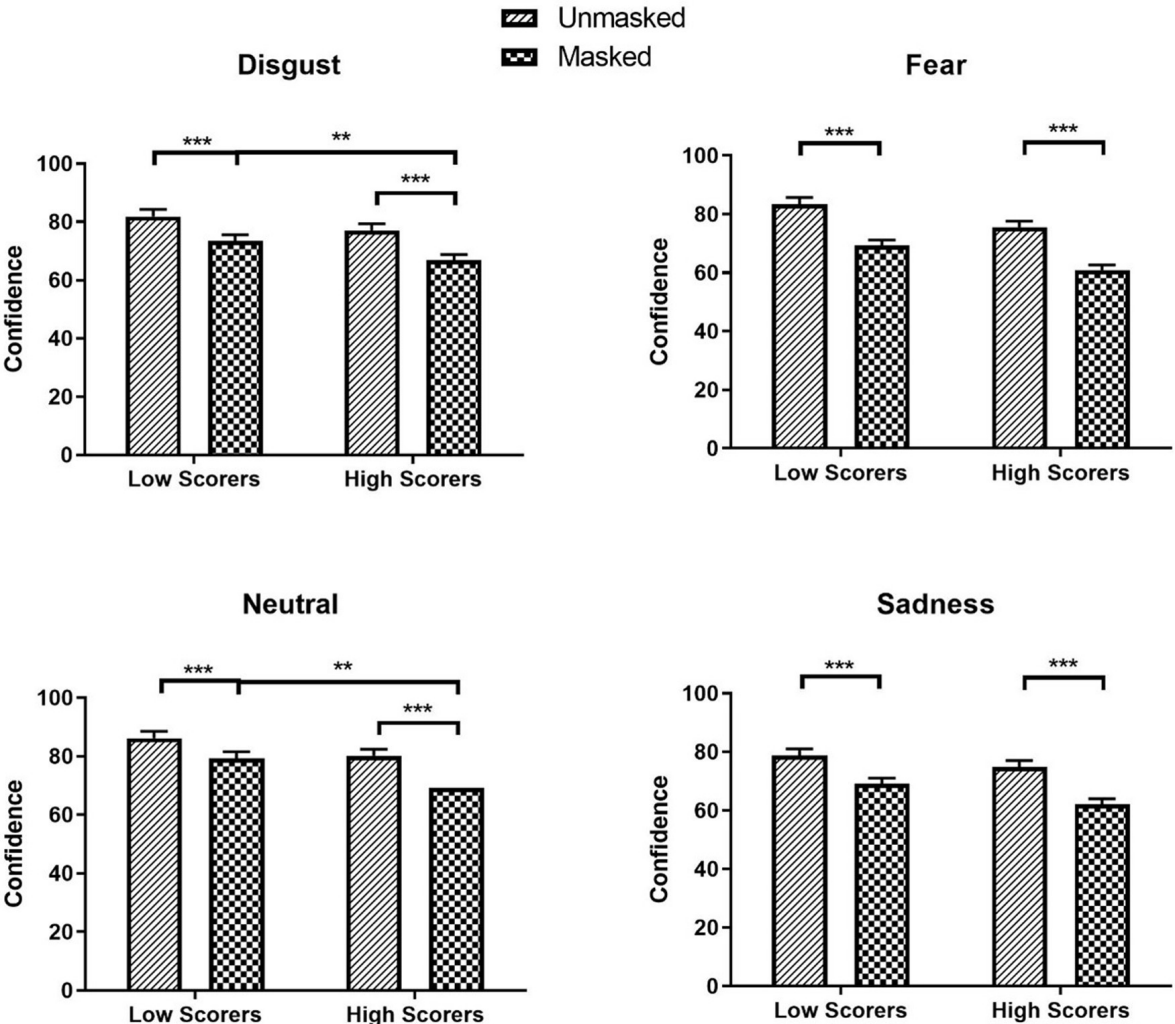
Confidence ratings for disgust, fear, neutral and sadness as a function of mask and AQ-10. * *p* < 0.05, ** *p* < 0.01, *** *p* < 0.001.

The significant main effects of Mask and AQ-10 were qualified by a significant Mask × AQ-10 interactions for disgust, fear, neutral, and sad faces (Disgust: *β* = 0.03, *SE* = 0.01, *χ²* (1) = 17.84, *z* = 4.22, *p* < .001; Fear: *β* = 0.03, *SE* = 0.01, *χ²* (1) = 24.31, *z* = 4.93, *p* < .001; Neutral: *β* = 0.06, *SE* = 0.01, *χ²* (1) = 89.46, *z* = 9.46, *p* < .001; Sadness: *β* = 0.05, *SE* = 0.01, *χ²* (1) = 58.65, *z* = 7.66, *p* < .001). High scorers were more confident in FER for disgust, fear, neutral, and sad unmasked faces (Disgust: *M* = 75.53, *SEM* = 2.11, 95% CI [71.51, 79.77]; Fear: *M* = 77.06, *SEM* = 2.30, 95% CI [72.69, 81.70]; Neutral: *M* = 80.15, *SEM* = 2.25, 95% CI [75.86, 84.69]; Sadness: *M* = 74.91, *SEM* = 2.14, 95% CI [70.83, 79.24]) compared to masked faces (Disgust: M = 68.97, SEM = 1.93, 95% CI [65.30, 72.85], *z* = -42.82, *p* < .001; Fear: M = 66.80, SEM = 1.99, 95% CI [63.00, 70.82], *z* = -29.23, *p* < .001; Neutral: M = 69.32, SEM = 1.95, 95% CI [65.60, 73.24], *z* = -30.33, *p* < .001; Sadness: M = 62.20, SEM = 1.78, 95% CI [58.80, 65.80], *z* = -37.09, *p* < .001). High scorers (Disgust: M = 60.90, SEM = 1.70, 95% CI [57.65, 64.33]; Neutral: M = 69.32, SEM = 1.95, 95% CI [65.60, 73.24]) were significantly less confident than low scorers (Disgust: M = 69.24, SEM = 1.93, 95% CI [65.56, 73.14], *z* = 3.25, *p* = .007; Neutral: M = 79.29, SEM = 2.23, 95% CI [75.04, 83.78], *z* = 3.38, *p* = .004) in identifying disgust and neutral masked emotions (Fig 6). No difference was observed between low and high scorers for confidence in disgust (*z* = 2.51; *p* = .072), fear (*z* = 1.43; *p* = .910), neutrality (*z* = 1.81; *p* = .425), and sadness (*z* = 1.25; *p* = .999) of unmasked face; and between low and high scorers for confidence in fear (*z* = 2.23; *p* = .156) and sad (*z* = 2.57; *p* = .062) masked faces.

**Fig 7:**
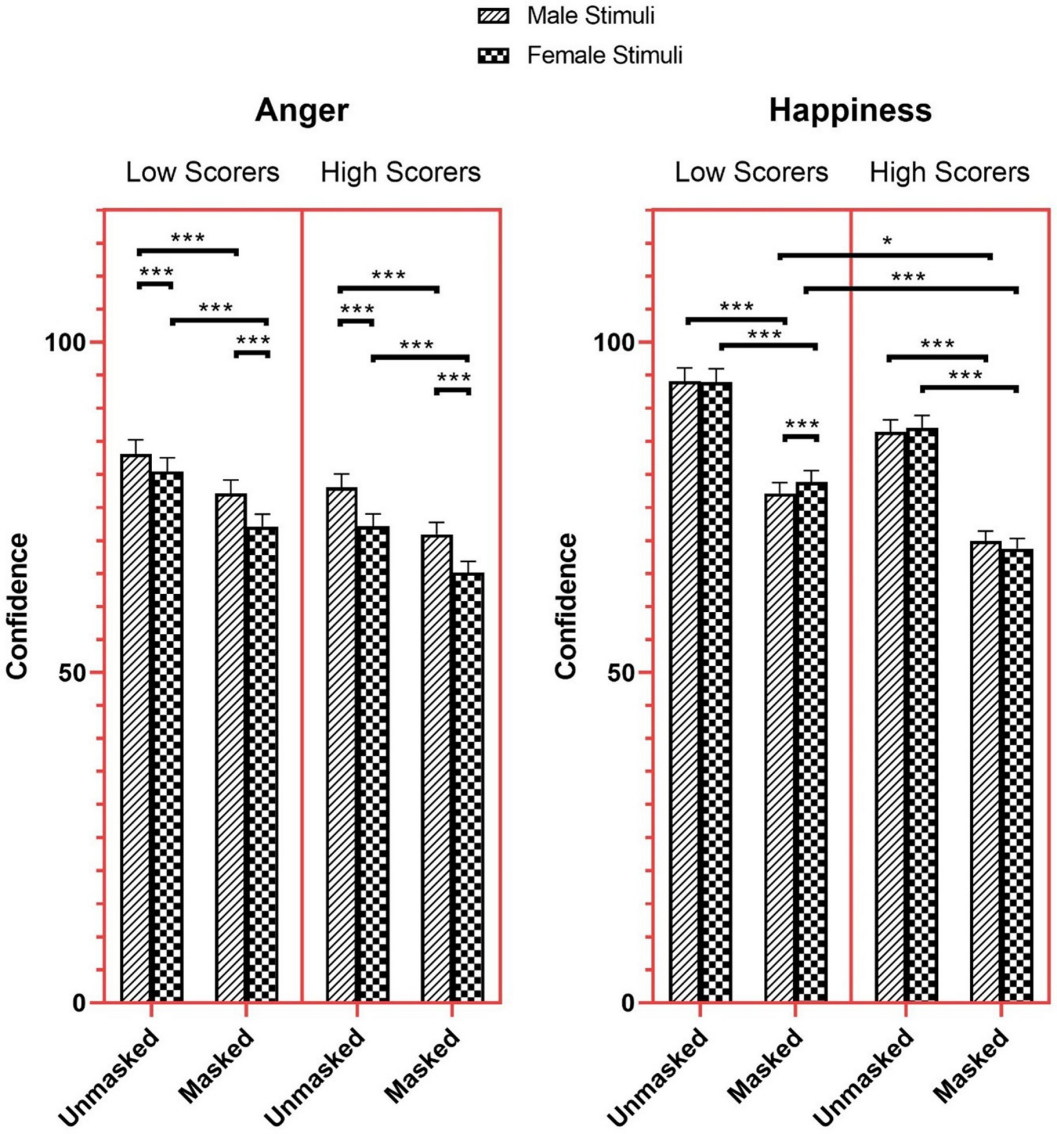
Confidence ratings for anger and happiness as a function of mask, AQ-10, and stimuli sex. * *p* < 0.05, ** *p* < 0.01, *** *p* < 0.001.

Results also showed a significant three-way Mask × AQ-10 × Stimulus Sex interaction for anger and happiness (Anger: *β* = -0.03, *SE* = 0.01, *χ²* (1) = 4.56, *z* = -2.14, *p* = .033; Happiness: *β* = 0.05, *SE* = 0.01, *χ²* (1) = 13.42, *z* = 3.66, *p* < .001). High scorers were more confident in identifying anger in masked male faces (*M* = 70.90, *SEM* = 1.84, 95% CI [67.37, 74.61]) compared to masked female faces (*M* = 65.14, *SEM* = 1.70, 95% CI [61.90, 68.56], *z* = 11.90, *p* < .001), but less confident compared to unmasked male faces (*M* = 78.03, *SEM* = 2.03, 95% CI [74.16, 82.11], *z* = -14.10, *p* < .001). High scorers were also more confident in anger recognition for unmasked female faces (*M* = 72.13, *SEM* = 1.88, 95% CI [68.54, 75.90]) compared to masked female faces (*z* = -14.38, *p* < .001), but less confident compared to unmasked male faces (*z* = 11.62, *p* < .001; Fig 7).

High scorers were less confident in identifying happiness in masked male faces (*M* = 69.90, *SEM* = 1.52, 95% CI [66.98, 72.95]) than happiness in unmasked male faces (*M* = 77.07, *SEM* = 1.68, 95% CI [73.85, 80.42], *z* = -31.69, *p* < .001). They were also less confident than low scorers’ (*M* = 86.39, *SEM* = 1.87, 95% CI [82.79, 90.14], *z* = 3.17, *p* = .043) in identifying happiness in masked male faces. Similarly, high scorers were less confident in identifying happiness in masked female faces (*M* = 68.75, *SEM* = 1.50, 95% CI [65.87, 71.75]) compared to happiness in unmasked female faces (*M* = 87.00, *SEM* = 1.89, 95% CI [83.38, 90.78], *z* = 4.45, *p* < .001), and less confident than low scorers (*M* = 78.84, *SEM* = 1.71, 95% CI [75.55, 82.27], *z* = -35.14, *p* < .001) in identifying happiness in masked female faces.

The results of post hoc comparison for the effect of masks on the degree of drop in FER confidence showed a greater reduction in confidence for fear, neutral, and sad faces for high scorers compared to low scorers. The mean difference in confidence ratings between masked and unmasked sad faces was 12.71 for high scorers, but only 9.78 for low scorers. Similarly, results show that high scorers’ mean difference between the two conditions for neutral faces was 10.83, but only 6.82 for low scorers. The reduction in confidence between unmasked and masked fearful faces for high scorers was 10.26, while only 8.48 for low scorers.

#### Intensity of expression

To investigate the effect of facial masks on the ratings of intensity of facial expressions, a series of generalized linear mixed models were conducted with each of the expressions as a fixed factor and participants as a random factor. Note that the question of expression intensity was not included for the neutral expression. Results showed significant main effects of Mask for intensity in all expressions (Anger: *β* = 0.05, *SE* = 0.01, *χ²* (1) = 232.45, *z* = 15.25, *p* < .001; Disgust: *β* = 0.16, *SE* = 0.01, *χ²* (1) = 2168.43, *z* = 46.57, *p* < .001; Fear: *β* = 0.08, *SE* = 0.01, *χ²* (1) = 545.44, *z* = 23.35, *p* < .001; Happiness: *β* = 0.28, *SE* = 0.01, *χ²* (1) = 6148.34, *z* = 78.41, *p* < .001; Sadness: *β* = 0.13, *SE* = 0.01, *χ²* (1) = 1291.58, *z* = 34.94, *p* < .001). Participants perceived anger under the masks (Anger: *M* = 66.90, *SEM* = 1.29, 95% CI [64.41, 69.48]) as less expressive (less intense) than without masks (Anger: *M* = 70.61, *SEM* = 1.36, 95% CI [67.98, 73.33]). Moreover, low scorers on the AQ-10 (Anger: *M* = 71.95, *SEM* = 1.96, 95% CI [68.22, 75.89]) perceived anger as more expressive than high scorers (Anger: *M* = 65.64, *SEM* = 1.79, 95% CI [62.23, 69.24], *β* = -0.09, *SE* = 0.04, *χ²* (1) = 5.68, *z* = -2.38, *p* = .017).

Additionally, the main effects of Stimulus Sex were significant for intensity of all expressions (Anger: *β* = -0.07, *SE* = 0.01, *χ²* (1) = 393.25, *z* = -19.38, *p* < .001; Disgust: *β* = -0.05, *SE* = 0.01, *χ²* (1) = 228.76, *z* = -15.12, *p* < .001; Fear: *β* = -0.04, *SE* = 0.01, *χ²* (1) = 134.50, *z* = -11.60, *p* < .001; Happiness: *β* = -0.03, *SE* = 0.01, *χ²* (1) = 67.71, *z* = -7.86, *p* < .001; Sadness: *β* = 0.06, *SE* = 0.01, *χ²* (1) = 299.27, *z* = 17.30, *p* < .001). Participants perceived male faces (Anger: *M* = 71.18, *SEM* = 1.37, 95% CI [68.54, 73.93]) as more expressive than female faces in anger (Anger: *M* = 66.36, *SEM* = 1.28, 95% CI [63.89, 68.92]).

**Fig 8:**
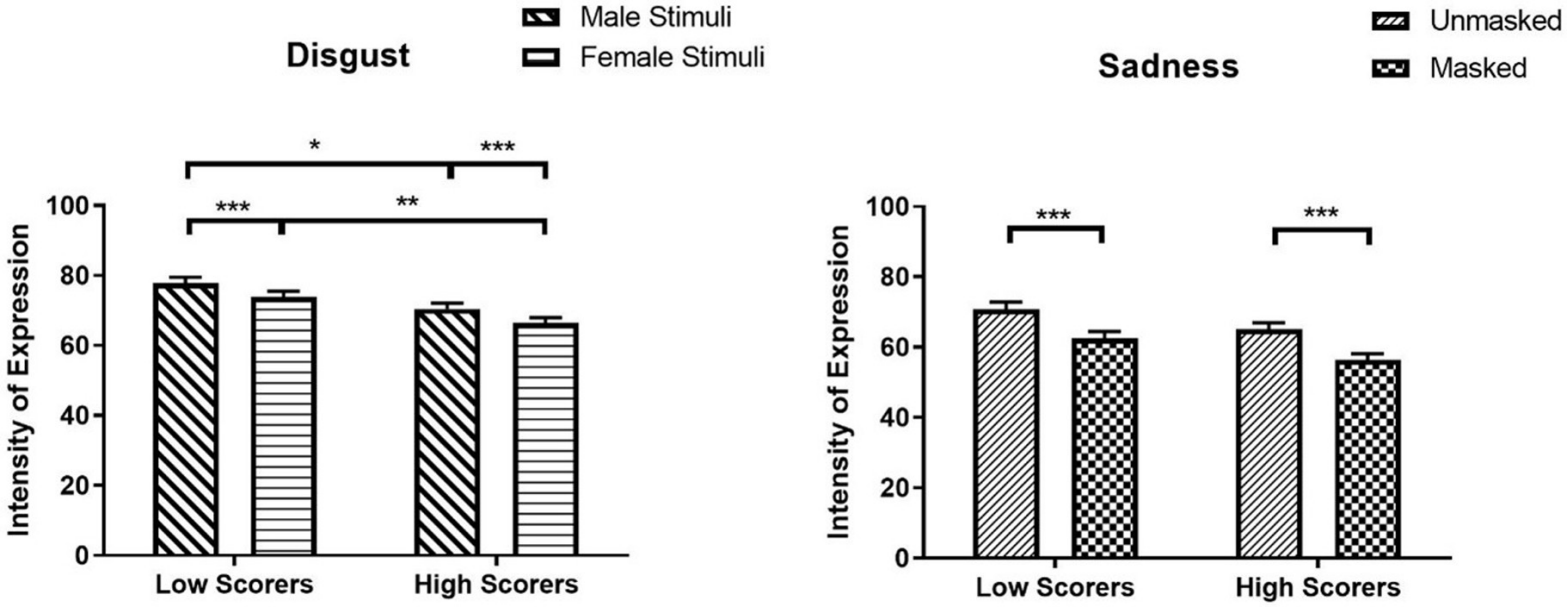
Intensity of expression ratings for disgust and sadness as a function of mask and AQ-10. * *p* < 0.05, ** *p* < 0.01, *** *p* < 0.001.

The significant main effects of Mask and AQ-10 were qualified by a significant Mask × AQ-10 interaction for sadness (*β* = 0.02, *SE* = 0.01, *χ²* (1) = 6.21, *z* = 2.49, *p* = .013). High scorers perceived sadness as more expressive in unmasked faces (*M* = 65.04, *SEM* = 1.92, 95% CI [61.39, 68.91]) compared to masked faces (*M* = 56.46, *SEM* = 1.67, 95% CI [53.29, 59.83], *z* = -26.64, *p* < .001; Fig 8). The AQ-10 × Stimulus Sex interaction was also significant for disgust (*β* = -0.01, *SE* = 0.01, *χ²* (1) = 4.37, *z* = -2.09, *p* = .037). High scorers perceived disgust in both male (*M* = 70.47, *SEM* = 1.67, 95% CI [67.27, 73.82]) and female faces (*M* = 66.37, *SEM* = 1.58, 95% CI [63.36, 69.53]) as less expressive compared to low scorers’ perception of disgust in male (*M* = 77.20, *SEM* = 1.83, 95% CI [73.70, 80.87], *z* = 2.72, *p* = .039) and female faces (*M* = 73.78, *SEM* = 1.75, 95% CI [70.43, 77.29], *z* = 3.15, *p* = .010), respectively. Also, high scorers perceived male faces as more expressive in disgust than female faces (*z* = 11.91, *p* < .001; Fig 8). No difference was observed between low and high scorers for confidence in sadness for both masked (*z* = 2.46; *p* = .083) and unmasked face (*z* = 2.02; *p* = .258).

**Fig 9:**
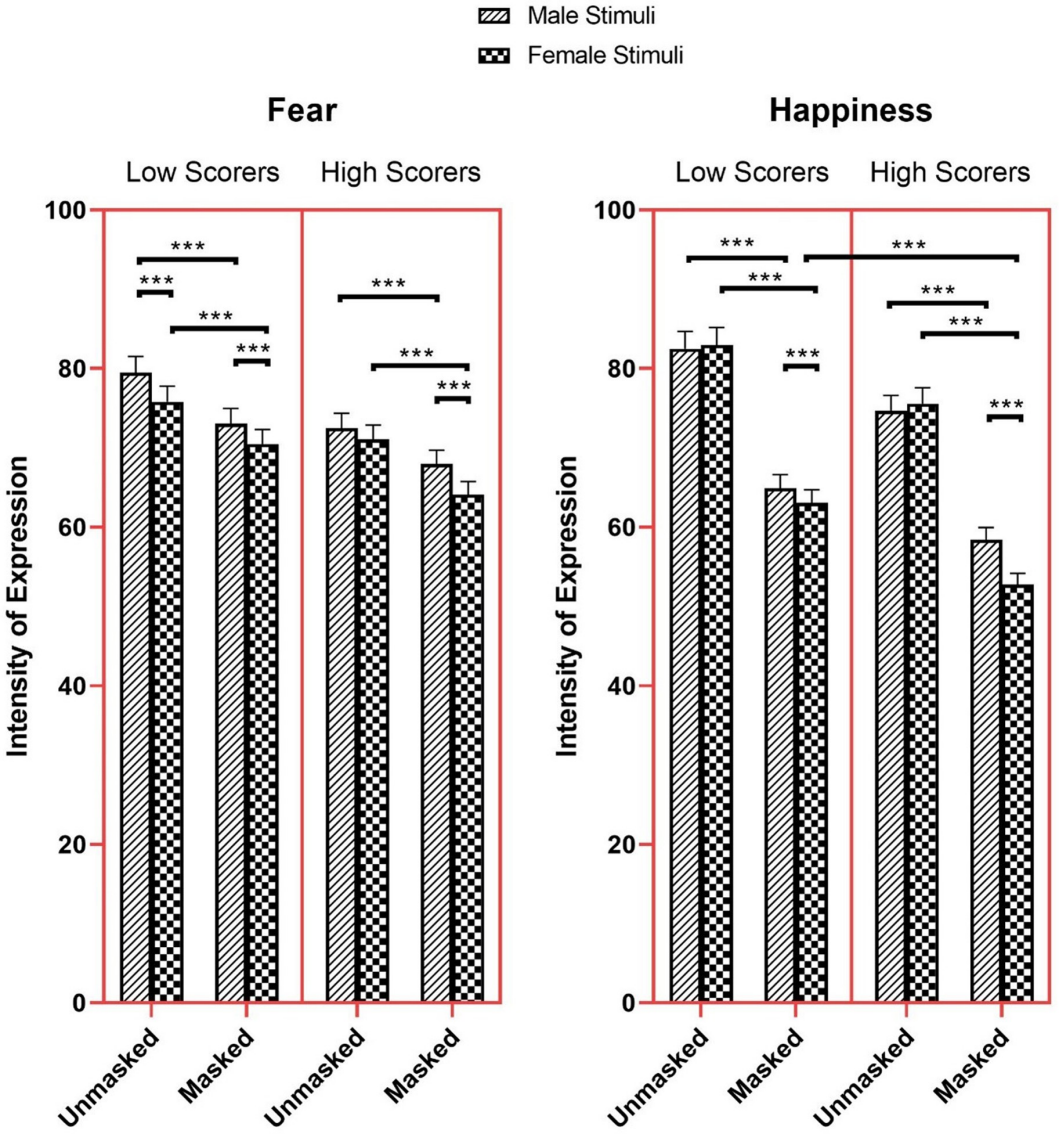
Intensity of expression ratings for fear and happiness as a function of mask, AQ-10 and stimuli sex. * *p* < 0.05, ** *p* < 0.01, *** *p* < 0.001.

The results also returned significant three-way Mask × AQ-10 × Stimulus Sex interactions for fear and happiness (Fear: *β* = 0.05, *SE* = 0.01, *χ²* (1) = 12.45, *z* = 3.53, *p* < .001; Happiness: *β* = 0.08, *SE* = 0.01, *χ²* (1) = 30.71, *z* = 5.54, *p* < .001). High scorers perceived fear in masked male face (*M* = 67.94, *SEM* = 1.75, 95% CI [64.59, 71.46]) as more expressive than masked female faces (*M* = 64.12, *SEM* = 1.65, 95% CI [60.96, 67.45], *z* = 8.02, *p* < .001), and less expressive than male unmasked faces (*M* = 72.49, *SEM* = 1.87, 95% CI [68.92, 76.24], *z* = -9.26, *p* < .001, Fig 9). Additionally, high scorers perceived fear in unmasked female faces (*M* = 71.05, *SEM* = 1.83, 95% CI [67.56, 74.73]) as more expressive than masked female face (*z* = -14.39, *p* < .001).

High scorers perceived happiness in female masked faces (*M* = 52.78, *SEM* = 1.41, 95% CI [50.10, 55.61]) as less expressive compared to low scorers (*M* = 63.07, *SEM* = 1.67, 95% CI [59.88, 66.44], *z* = 4.74, *p* < .001). Moreover, high scorers perceived happiness in female masked faces as less expressive than female unmasked faces (*M* = 75.56, *SEM* = 2.00, 95% CI [71.75, 79.58], *z* = -48.33, *p* < .001) and male masked faces (*M* = 58.42, *SEM* = 1.55, 95% CI [55.45, 61.54], *z* = 12.90, *p* < .001, Fig 9). High scorers also perceived masked male faces as less expressive in happiness than unmasked male faces (*M* = 74.64, *SEM* = 1.97, 95% CI [70.86, 78.61], *z* = 33.89, *p* < .001). For histogram and density plot of the confidence and expression intensity data as a function of AQ-10, Mask, and Stimulus Sex see S1 File.

### Discussion

Testing the effect of facial mask on FER in low and high AQ-10 scorers showed that high scorers (those who endorsed more autistic traits) had less accuracy in FER than low scorers (those who endorsed few autistic traits), which supports previous research (deficits in identifying anger, disgust, fear [30, 51], sadness, surprise [30], and happiness [51]). Additionally, high scorers were less confident than low scorers in recognizing emotions labeled as anger, disgust, happiness, and neutral in unmasked faces, while they were less confident in recognizing masked faces labeled as disgust and neutral compared to low scorers. Furthermore, high scorers rated all of the emotions as less intense than did low scorers. They also rated male faces displaying disgust and fear, as well as masked female faces displaying happiness, as less intense than did low scorers.

Similar to the overall results in the previous experiments, high scorers on the AQ-10 were better at FER in male faces than in female faces, and were more confident in the recognition of anger in male faces compared to anger in female faces. While both high and low scorers’ confidence dropped significantly from unmasked to masked emotions of fear, neutral, and sad faces, the drop in confidence trended towards being larger for high scorers. Additionally, similar to low scorers, high scorers performed best when identifying unmasked faces labeled as happy. This supports previous reports that people with ASD are most accurate when identifying faces expressing happiness [30].

Recall that we hypothesised that high scorers on the AQ-10 would show deficits in FER when compared to low scorers. The results showed this to be the case. However, we also predicted that the introduction of masks would impede FER in high scorers more than they would in low scorers. This was not supported by the data. This lack of support is unexpected given that prior research has found that people use the mouth to identify happiness [52] and disgust [9], the eyes to discern fear and sadness [9], and that individuals with ASD look less at the eyes of fearful or neutral faces [53]. While masks caused a greater drop in confidence for high scorers than low scorers in expressions labeled as fear, neutral, and sad, there was no significant interaction between AQ-10 scores and masks for FER, despite high scorers rating both the intensity of emotions and their confidence in their FER ability lower than low scorers.

## General discussion

Since the outbreak of COVID-19, health organizations have encouraged the general public to wear facial masks in order to curb its spread. While masks help combat against infection, they also hinder day-to-day social interactions, with previously unknown impact on individuals with autistic traits. As these individuals have been shown to have deficits in reading the emotions of others, the introduction of masks in daily social interactions was anticipated to have a greater impact on them than neurotypicals (NTs). Across three experiments we investigated the influence of masks on facial emotion recognition (FER). We tested the effect of masks on recognition of anger, disgust, fear, happiness, neutrality, and sadness in male and female faces using different samples (university students in Experiment 1, and the general population in Experiment 2), as well as examined the role of autistic traits (scores on the 10-item Autism Spectrum Quotient). We also assessed the confidence of participants in their judgements, and their perceived intensity of facial expressions. Overall our results showed that the introduction of masks disrupted participants’ FER ability, their confidence in judging facial expressions, and their perception of emotion intensity.

Participants’ accuracy in expression recognition decreased when faces were masked, a finding that was observed across all three studies, supporting previous research that reported similar findings [18, 21, 22]. Participants in the current study were also more accurate in FER when categorizing male versus female faces, although participants in Experiment 2 showed this pattern only in the masked condition.

Masks also affected confidence in FER. In all three experiments, participants were less confident in their judgements for all emotions in the mask condition compared to the unmasked condition, results that dovetail with the recent work by Carbon [22]. In the current study, participants were also less confident in identifying anger in female masked faces than male masked faces. For Experiments 1 and 2, participants were also less confident in identifying sadness in masked male faces than in masked female faces.

Because recent work has not addressed the effect that masks may have on the perception of emotional intensity, we asked participants to rate the level of expression (or intensity) in faces with and without masks. Across the three experiments, masks resulted in all facial expressions being perceived as less intense. These findings extend the research on the impact of wearing masks, beyond disruptions in FER accuracy and confidence. Sex differences in perception of expression intensity were also found: Experiments 1 and 2 returned higher ratings of sadness in masked female faces compared to masked male faces, and more intense perceptions of happiness for masked male faces than masked female faces. Additionally, participants in Experiment 2 perceived a higher intensity of anger in masked male faces than in masked female faces, while participants in Experiment 1 rated fear as more intense in masked male faces than in masked female faces.

Finally, Experiment 3 examined how traits of autism are related to perceptions of emotion expression. Recent research on COVID-19’s impact on the autistic population has seen a focus on areas such as disruption of routines or services, how caregivers coped during lockdown, and mask tolerance training [54–59]. To extend the ASD and COVID-19-related research, we examined the effects of masks on possible social interactions for individuals with autistic traits in terms of FER, participants’ confidence in their FER abilities, and their perception of facial emotion intensity.

Our results showed that compared to low scorers (participants who scored 5 or lower on the AQ-10), high scorers (those who scored 6 or higher) were significantly less accurate overall in FER, supporting findings from previous research [30]. However, we found no significant interaction between AQ-10 scores and masks. Given that masks cover the bottom half of the face, this result is counter to previous research that showed individuals with autistic traits preferentially look towards the mouth when viewing faces [35, 36, but see 33], and that they rely more on the mouth region than NTs for categorizing emotions [38]. One possible explanation for our results may be rooted in Social Motivation Theory (SMT; [60]), which proposes that individuals with autistic traits can attend to salient social cues when prompted to do so, or if they believe doing so can help complete a task. The wearing of a mask, then, could serve as a visual prompt for autistic individuals to actively search for emotional clues elsewhere on the face.

To the best of our knowledge, this is the first study to examine the effect of facial masks on intensity ratings of expressions in autistic individuals’ perception of emotion in the faces of others. However, our finding that high scorers rated the expressions in the stimuli as significantly less intense than low scorers does align with previous findings that autistic individuals have difficulty with rating emotions in unmasked faces [61]. Perceiving emotions as less intense, and therefore less informative, also aligns with our additional finding that high scorers reported being significantly less confident than low scorers in their ability to recognize anger, disgust, happiness, and neutral emotions in faces. Extending the research on autistic individuals’ confidence in FER, we found their confidence trended lower for masked fear, neutral, and sad faces. We are unaware of previous studies examining autistic individuals’ confidence in their FER abilities while viewing masked faces, although our results support Bekele et al. [62] who found autistic adolescents were less confident than controls (but similarly accurate) in FER in tasks involving unmasked faces. Note, however, that Sawyer et al. [63], found autistic participants to be similarly confident to controls (but less accurate) in FER tasks that also involved unmasked faces. This apparent discrepancy appears to reflect the Dunning-Kruger effect, whereby a person can either over-estimate or under-estimate their ability at a task [64]. The bias that is expressed by those who trend towards autism seems to depend on the aspect of the task that is emphasised. When performance is emphasised—as in our study and that of Bekele et al. [62]—participants underestimate their competence (i.e., their confidence is below average). And when confidence is emphasised, as in Sawyer et al. [63] who instructed participants to choose the level that balanced speed and accuracy, participants overestimate their competence (i.e., their performance is below average).

As in the first two experiments, high scorers in Experiment 3 were less confident in identifying anger in masked female faces than in masked male faces, and high scores rated happiness in masked male faces as more intense than in masked female faces. High scorers were also less confident than low scorers in categorizing happiness in both male and female masked faces. Similar to Experiment 1, high scorers rated fear as more intense in masked male faces than in masked female faces. Thus, while the introduction of masks did not hinder FER accuracy significantly more for high scorers on the AQ-10, high scorers’ confidence in emotion recognition and perception of emotional intensity for certain emotions were impacted by masks more than low scorers. Confidence was particularly reduced for high scorers when viewing masked faces labeled as disgust, happy, or neutral, whereas female masked faces labeled as happiness, and disgust were rated as significantly less intense.

In summary, our results support previous research showing that wearing facial masks decreases both facial expression recognition [18, 21, 22], and confidence in expression identification [22]. Contrary to previous research that found no effects of facial masks on recognition of fear [22], neutrality [18, 21, 22] or sadness [21] our studies showed that facial masks disrupt recognition of all investigated emotions. We also had the novel finding that perception of expression intensity of anger, disgust, fear, happiness, neutrality, and sadness were reduced by masks. Our novel finding that high AQ-10 scorers were lower in FER accuracy, FER confidence, and emotion perception relative to low AQ-10 scorers when viewing masked faces adds to the extant COVID-19 autism-related research, as well as to the broader FER and ASD research. These novel findings are consistent with previous research findings that people with autistic traits have greater difficulty than NTs in FER [30, 65, 66]. Our results suggest that the introduction of mask wearing in the current pandemic climate will reduce confidence in people with autistic traits in FER, particularly when attempting to discern fearful, neutral, or happy faces. The current investigation also extends the previous research by showing that masks affect all expressions in terms of recognition, confidence in identification, and intensity perception. Moreover, this research contributes to the literature by confirming these effects in individuals with autistic traits and comparing them with neurotypicals.

Possible limitations in the current study include that reaction times were not recorded. While there were no significant differences between the two AQ-10 groups in FER accuracy for masked faces, it is not possible to determine whether or not high scorers took longer to label facial expressions. As autistic individuals can take longer to process faces [67, 68], future studies should examine potential differences in response times between individuals with autistic traits and neurotypicals.

Another limitation was that our participants were predominantly female for all three studies. Had there been a better balance between male and female participants, we could have explored differences between males and females. Additionally, the images used in the study exhibited emotions at their peak intensity, which is not always the case during natural social interactions. Given that high scorers reported the expressions in the stimuli as less intense than low scorers did, their confidence and accuracy might be further reduced when interacting with others in day-to-day social situations when expressions might not be delivered at peak intensity, with or without masks. Moreover, all three experiments were conducted online, and while online experiments are shown to be reliable and have external validity [69], we suggest that future research investigate the effect of facial masks in FER by testing individuals who score high and low on the AQ-10 in person. Also, as dynamic stimuli can facilitate emotion perception [42, 43], we suggest it would be instructive to examine the effect of facial masks on FER when NTs and autistic individuals are presented with dynamic versus static images.

In conclusion, across three experiments in which we tested individuals from different populations and with different levels of autistic traits, we found that facial masks have a negative effect on facial expression recognition. Wearing facial masks also reduces the intensity of the emotion that is being perceived and observers’ confidence in their ability to correctly identify the emotion, particularly so for those with autistic traits.

## Supporting information

S1 File S1-S3 Tables and histogram and density plots. (DOCX)
