# Supplementary material for: Facial masks affect emotion recognition in the general population and individuals with autistic traits
Source: PLoS One. 2021 Sep 30;16(9):e0257740. doi: 10.1371/journal.pone.0257740 (PMC8483373; doi:10.1371/journal.pone.0257740)

| Table S1. Frequency and Percentage of Responses for Facial Expressions, by Stimuli Sex and Presence/Absence of Facial Masks (Study 1) | | | | | | | | | | | | | | | | | | | | | | | | | | |
| --- | --- | --- | --- | --- | --- | --- | --- | --- | --- | --- | --- | --- | --- | --- | --- | --- | --- | --- | --- | --- | --- | --- | --- | --- | --- | --- |
|  |  | Without Mask | | | | | | | | | | | |  | With Mask | | | | | | | | | | | |
|  |  | Anger | | Disgust | | Fear | | Happy | | Neutral | | Sad | |  | Anger | | Disgust | | Fear | | Happy | | Neutral | | Sad | |
| Stimuli Sex | Expression | *n* | *%* | *n* | *%* | *n* | *%* | *n* | *%* | *n* | *%* | *n* | *%* |  | *n* | *%* | *n* | *%* | *n* | *%* | *n* | *%* | *n* | *%* | *n* | *%* |
| Male | Anger | 2757 | 82.1 | 220 | 6.5 | 32 | 1.0 | 11 | 0.3 | 80 | 2.4 | 32 | 1.0 |  | 3212 | 85.0 | 1072 | 28.4 | 60 | 1.6 | 39 | 1.0 | 56 | 1.5 | 145 | 3.8 |
|  | Disgust | 320 | 9.5 | 2930 | 87.2 | 243 | 7.2 | 22 | 0.7 | 27 | 0.8 | 757 | 22.5 |  | 230 | 6.1 | 2064 | 54.6 | 202 | 5.3 | 58 | 1.5 | 24 | 0.6 | 647 | 17.1 |
|  | Fear | 31 | 0.9 | 72 | 2.1 | 3007 | 89.5 | 13 | 0.4 | 15 | 0.4 | 240 | 7.1 |  | 48 | 1.3 | 153 | 4.0 | 3277 | 86.7 | 42 | 1.1 | 34 | 0.9 | 377 | 10.0 |
|  | Happy | 18 | 0.5 | 15 | 0.4 | 23 | 0.7 | 3265 | 97.2 | 13 | 0.4 | 27 | 0.8 |  | 16 | 0.4 | 73 | 1.9 | 162 | 4.3 | 3311 | 87.6 | 57 | 1.5 | 285 | 7.5 |
|  | Neutral | 32 | 1.0 | 25 | 0.7 | 38 | 1.1 | 30 | 0.9 | 3133 | 93.2 | 49 | 1.5 |  | 46 | 1.2 | 48 | 1.3 | 30 | 0.8 | 188 | 5.0 | 3500 | 92.6 | 257 | 6.8 |
|  | Sad | 202 | 6.0 | 98 | 2.9 | 17 | 0.5 | 19 | 0.6 | 92 | 2.7 | 2255 | 67.1 |  | 228 | 6.0 | 370 | 9.8 | 49 | 1.3 | 142 | 3.8 | 109 | 2.9 | 2069 | 54.7 |
| Female | Anger | 2426 | 72.2 | 520 | 15.5 | 65 | 1.9 | 8 | 0.2 | 169 | 5 | 68 | 2 |  | 1994 | 67.8 | 1013 | 34.5 | 57 | 1.9 | 23 | 0.8 | 56 | 1.9 | 77 | 2.6 |
|  | Disgust | 529 | 15.7 | 2647 | 78.8 | 233 | 6.9 | 19 | 0.6 | 48 | 1.4 | 287 | 8.5 |  | 410 | 13.9 | 1006 | 34.2 | 104 | 3.5 | 21 | 0.7 | 18 | 0.6 | 135 | 4.6 |
|  | Fear | 54 | 1.6 | 39 | 1.2 | 2983 | 89 | 11 | 0.3 | 38 | 1.1 | 240 | 7.1 |  | 65 | 2.2 | 65 | 2.2 | 2570 | 87.4 | 34 | 1.2 | 40 | 1.4 | 192 | 6.5 |
|  | Happy | 16 | 0.5 | 13 | 0.4 | 20 | 0.6 | 3260 | 97 | 27 | 0.8 | 26 | 0.8 |  | 21 | 0.7 | 226 | 7.7 | 104 | 3.5 | 2212 | 75.2 | 83 | 2.8 | 21 | 0.7 |
|  | Neutral | 31 | 0.9 | 26 | 0.8 | 31 | 0.9 | 38 | 1.1 | 2858 | 85 | 69 | 2.1 |  | 49 | 1.7 | 32 | 1.1 | 74 | 2.5 | 417 | 14.2 | 2562 | 87.1 | 87 | 3.0 |
|  | Sad | 304 | 9 | 115 | 3.4 | 28 | 0.8 | 24 | 0.7 | 220 | 6.5 | 2670 | 80 |  | 401 | 13.6 | 598 | 20.3 | 31 | 1.1 | 233 | 7.9 | 181 | 6.2 | 2428 | 82.6 |

| Table S2. Frequency and Percentage of Responses for Facial Expressions, by Stimuli Sex and Presence/Absence of Facial Masks (Study 2) | | | | | | | | | | | | | | | | | | | | | | | | | | |
| --- | --- | --- | --- | --- | --- | --- | --- | --- | --- | --- | --- | --- | --- | --- | --- | --- | --- | --- | --- | --- | --- | --- | --- | --- | --- | --- |
|  |  | Without Mask | | | | | | | | | | | |  | With Mask | | | | | | | | | | | |
|  |  | Anger | | Disgust | | Fear | | Happy | | Neutral | | Sad | |  | Anger | | Disgust | | Fear | | Happy | | Neutral | | Sad | |
| Stimulus sex | Expression | *n* | *%* | *n* | *%* | *n* | *%* | *n* | *%* | *n* | *%* | *n* | *%* |  | *n* | *%* | *n* | *%* | *n* | *%* | *n* | *%* | *n* | *%* | *n* | *%* |
| Female | Anger | 782 | 65.5 | 221 | 18.5 | 142 | 12 | 12 | 1 | 43 | 3.6 | 66 | 5.5 |  | 709 | 59 | 413 | 35 | 137 | 12 | 57 | 4.8 | 56 | 4.7 | 92 | 7.7 |
|  | Disgust | 199 | 16.7 | 744 | 62.3 | 169 | 14 | 4 | 0.3 | 14 | 1.2 | 67 | 5.6 |  | 144 | 12 | 311 | 26 | 80 | 6.7 | 18 | 1.5 | 13 | 1.1 | 43 | 3.6 |
|  | Fear | 62 | 5.2 | 83 | 7 | 808 | 68 | 11 | 0.9 | 39 | 3.3 | 130 | 11 |  | 89 | 7.5 | 70 | 5.9 | 883 | 74 | 40 | 3.4 | 61 | 5.1 | 144 | 12 |
|  | Happy | 37 | 3.1 | 14 | 1.2 | 13 | 1.1 | 1145 | 96 | 37 | 3.1 | 17 | 1.4 |  | 35 | 2.9 | 103 | 8.6 | 37 | 3.1 | 765 | 64 | 107 | 9 | 38 | 3.2 |
|  | Neutral | 19 | 1.6 | 4 | 0.3 | 12 | 1 | 5 | 0.4 | 887 | 74 | 45 | 3.8 |  | 23 | 1.9 | 29 | 2.4 | 18 | 1.5 | 195 | 16 | 824 | 69 | 63 | 5.3 |
|  | Sad | 95 | 8 | 128 | 10.7 | 50 | 4.2 | 17 | 1.4 | 174 | 15 | 869 | 73 |  | 194 | 16 | 268 | 22 | 39 | 3.3 | 119 | 10 | 133 | 11 | 814 | 68 |
| Male | Anger | 835 | 69.9 | 117 | 9.8 | 118 | 9.9 | 16 | 1.3 | 48 | 4 | 56 | 4.7 |  | 877 | 74 | 340 | 29 | 117 | 9.8 | 69 | 5.8 | 60 | 5 | 130 | 11 |
|  | Disgust | 146 | 12.2 | 831 | 69.6 | 172 | 14 | 6 | 0.5 | 10 | 0.8 | 147 | 12 |  | 65 | 5.4 | 433 | 36 | 76 | 6.4 | 43 | 3.6 | 16 | 1.3 | 121 | 10 |
|  | Fear | 52 | 4.4 | 75 | 6.3 | 813 | 68 | 14 | 1.2 | 20 | 1.7 | 132 | 11 |  | 50 | 4.2 | 95 | 8 | 907 | 76 | 40 | 3.4 | 41 | 3.4 | 113 | 9.5 |
|  | Happy | 10 | 0.8 | 12 | 1 | 18 | 1.5 | 1144 | 96 | 32 | 2.7 | 17 | 1.4 |  | 29 | 2.4 | 62 | 5.2 | 39 | 3.3 | 872 | 73 | 94 | 7.9 | 129 | 11 |
|  | Neutral | 14 | 1.2 | 6 | 0.5 | 7 | 0.6 | 3 | 0.3 | 957 | 80 | 19 | 1.6 |  | 24 | 2 | 15 | 1.3 | 17 | 1.4 | 55 | 4.6 | 865 | 72 | 115 | 9.6 |
|  | Sad | 137 | 11.5 | 153 | 12.8 | 66 | 5.5 | 11 | 0.9 | 127 | 11 | 823 | 69 |  | 149 | 13 | 249 | 21 | 38 | 3.2 | 115 | 9.6 | 118 | 9.9 | 586 | 49 |

| Table S3. Frequency and Percentage of Responses for Facial Expressions, for Participants differing in AQ-10, and stimuli differing in Sex and Presence/Absence of Facial Masks (Study 3) | | | | | | | | | | | | | | | | | | | | | | | | | | | |
| --- | --- | --- | --- | --- | --- | --- | --- | --- | --- | --- | --- | --- | --- | --- | --- | --- | --- | --- | --- | --- | --- | --- | --- | --- | --- | --- | --- |
|  |  |  | Without Mask | | | | | | | | | | | |  | With Mask | | | | | | | | | | | |
|  |  |  | Anger | | Disgust | | Fear | | Happy | | Neutral | | Sad | |  | Anger | | Disgust | | Fear | | Happy | | Neutral | | Sad | |
| AQ-10 | Stimulus sex | Expression | *n* | *%* | *n* | *%* | *n* | *%* | *n* | *%* | *n* | *%* | *n* | *%* |  | *n* | *%* | *n* | *%* | *n* | *%* | *n* | *%* | *n* | *%* | *n* | *%* |
| Low Scorers | Male | Anger | 484 | 85.2 | 34 | 6.0 | 4 | 0.7 | 0 | 0 | 21 | 3.7 | 4 | 0.7 |  | 495 | 87.1 | 186 | 32.7 | 8 | 1.4 | 3 | 0.5 | 2 | 0.4 | 17 | 3.0 |
|  |  | Disgust | 45 | 7.9 | 515 | 90.7 | 37 | 6.5 | 0 | 0 | 1 | 0.2 | 132 | 23.2 |  | 44 | 7.7 | 290 | 51.1 | 32 | 5.6 | 3 | 0.5 | 0 | 0 | 95 | 16.7 |
|  |  | Fear | 3 | 0.5 | 8 | 1.4 | 517 | 91.0 | 0 | 0 | 1 | 0.2 | 49 | 8.6 |  | 3 | 0.5 | 20 | 3.5 | 507 | 89.3 | 3 | 0.5 | 2 | 0.4 | 77 | 13.6 |
|  |  | Happy | 1 | 0.2 | 0 | 0 | 8 | 1.4 | 565 | 99.5 | 2 | 0.4 | 4 | 0.7 |  | 0 | 0 | 5 | 0.9 | 17 | 3.0 | 513 | 90.3 | 1 | 0.2 | 44 | 7.7 |
|  |  | Neutral | 5 | 0.9 | 1 | 0.2 | 2 | 0.4 | 3 | 0.5 | 532 | 93.7 | 2 | 0.4 |  | 1 | 0.2 | 5 | 0.9 | 3 | 0.5 | 27 | 4.8 | 553 | 97.4 | 46 | 8.1 |
|  |  | Sad | 30 | 5.3 | 10 | 1.8 | 0 | 0 | 0 | 0 | 11 | 1.9 | 377 | 66.4 |  | 25 | 4.4 | 62 | 10.9 | 1 | 0.2 | 19 | 3.3 | 10 | 1.8 | 289 | 50.9 |
|  | Female | Anger | 432 | 76.1 | 81 | 14.3 | 12 | 2.1 | 0 | 0 | 29 | 5.1 | 9 | 1.6 |  | 430 | 75.7 | 189 | 33.3 | 8 | 1.4 | 0 | 0 | 12 | 2.1 | 26 | 4.6 |
|  |  | Disgust | 69 | 12.1 | 475 | 83.6 | 30 | 5.3 | 1 | 0.2 | 2 | 0.4 | 44 | 7.7 |  | 67 | 11.8 | 236 | 41.5 | 21 | 3.7 | 1 | 0.2 | 2 | 0.4 | 33 | 5.8 |
|  |  | Fear | 2 | 0.4 | 3 | 0.5 | 523 | 92.1 | 1 | 0.2 | 3 | 0.5 | 47 | 8.3 |  | 4 | 0.7 | 13 | 2.3 | 518 | 91.2 | 3 | 0.5 | 6 | 1.1 | 39 | 6.9 |
|  |  | Happy | 1 | 0.2 | 2 | 0.4 | 1 | 0.2 | 563 | 99.1 | 5 | 0.9 | 0 | 0 |  | 2 | 0.4 | 37 | 6.5 | 14 | 2.5 | 460 | 81.0 | 12 | 2.1 | 0 | 0 |
|  |  | Neutral | 6 | 1.1 | 2 | 0.4 | 2 | 0.4 | 2 | 0.4 | 498 | 87.7 | 8 | 1.4 |  | 8 | 1.4 | 4 | 0.7 | 7 | 1.2 | 70 | 12.3 | 508 | 89.4 | 7 | 1.2 |
|  |  | Sad | 58 | 10.2 | 5 | 0.9 | 0 | 0 | 1 | 0.2 | 31 | 5.5 | 460 | 81.0 |  | 57 | 10.0 | 89 | 15.7 | 0 | 0 | 34 | 6.0 | 28 | 4.9 | 463 | 81.5 |
| High Scorers | Male | Anger | 451 | 79.4 | 38 | 6.7 | 8 | 1.4 | 2 | 0.4 | 23 | 4.0 | 6 | 1.1 |  | 493 | 86.8 | 182 | 32.0 | 15 | 2.6 | 4 | 0.7 | 9 | 1.6 | 23 | 4.0 |
|  |  | Disgust | 57 | 10.0 | 488 | 85.9 | 44 | 7.7 | 4 | 0.7 | 6 | 1.1 | 133 | 23.4 |  | 28 | 4.9 | 292 | 51.4 | 29 | 5.1 | 14 | 2.5 | 3 | 0.5 | 97 | 17.1 |
|  |  | Fear | 6 | 1.1 | 9 | 1.6 | 500 | 88.0 | 5 | 0.9 | 0 | 0 | 36 | 6.3 |  | 9 | 1.6 | 21 | 3.7 | 493 | 86.8 | 6 | 1.1 | 4 | 0.7 | 47 | 8.3 |
|  |  | Happy | 8 | 1.4 | 5 | 0.9 | 8 | 1.4 | 553 | 97.4 | 3 | 0.5 | 4 | 0.7 |  | 2 | 0.4 | 13 | 2.3 | 19 | 3.3 | 482 | 84.9 | 7 | 1.2 | 48 | 8.5 |
|  |  | Neutral | 4 | 0.7 | 5 | 0.9 | 4 | 0.7 | 3 | 0.5 | 519 | 91.4 | 11 | 1.9 |  | 3 | 0.5 | 7 | 1.2 | 7 | 1.2 | 30 | 5.3 | 530 | 93.3 | 41 | 7.2 |
|  |  | Sad | 42 | 7.4 | 23 | 4.0 | 4 | 0.7 | 1 | 0.2 | 17 | 3.0 | 378 | 66.5 |  | 33 | 5.8 | 53 | 9.3 | 5 | 0.9 | 32 | 5.6 | 15 | 2.6 | 312 | 54.9 |
|  | Female | Anger | 375 | 66.0 | 97 | 17.1 | 18 | 3.2 | 1 | 0.2 | 27 | 4.8 | 7 | 1.2 |  | 388 | 68.3 | 158 | 27.8 | 9 | 1.6 | 8 | 1.4 | 12 | 2.1 | 18 | 3.2 |
|  |  | Disgust | 110 | 19.4 | 432 | 76.1 | 27 | 4.8 | 2 | 0.4 | 10 | 1.8 | 44 | 7.7 |  | 91 | 16.0 | 229 | 40.3 | 34 | 6.0 | 3 | 0.5 | 8 | 1.4 | 37 | 6.5 |
|  |  | Fear | 11 | 1.9 | 7 | 1.2 | 506 | 89.1 | 5 | 0.9 | 8 | 1.4 | 47 | 8.3 |  | 11 | 1.9 | 17 | 3.0 | 489 | 86.1 | 7 | 1.2 | 5 | 0.9 | 35 | 6.2 |
|  |  | Happy | 4 | 0.7 | 7 | 1.2 | 2 | 0.4 | 550 | 96.8 | 3 | 0.5 | 4 | 0.7 |  | 5 | 0.9 | 55 | 9.7 | 14 | 2.5 | 414 | 72.9 | 13 | 2.3 | 3 | 0.5 |
|  |  | Neutral | 3 | 0.5 | 2 | 0.4 | 5 | 0.9 | 4 | 0.7 | 485 | 85.4 | 15 | 2.6 |  | 11 | 1.9 | 6 | 1.1 | 16 | 2.8 | 86 | 15.1 | 494 | 87.0 | 20 | 3.5 |
|  |  | Sad | 65 | 11.4 | 23 | 4.0 | 10 | 1.8 | 6 | 1.1 | 35 | 6.2 | 451 | 79.4 |  | 62 | 10.9 | 103 | 18.1 | 6 | 1.1 | 50 | 8.8 | 36 | 6.3 | 455 | 80.1 |

**Confidence_Anger**


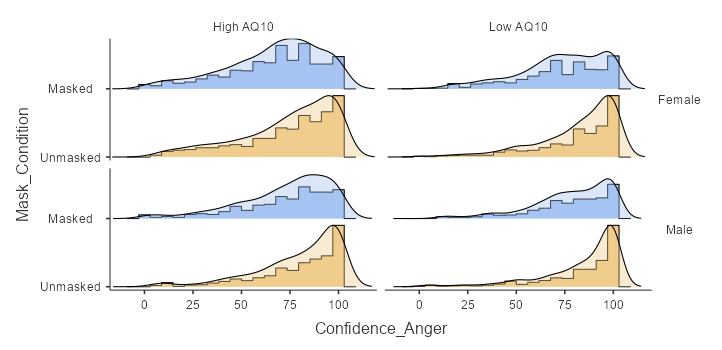


**Expression_Anger**


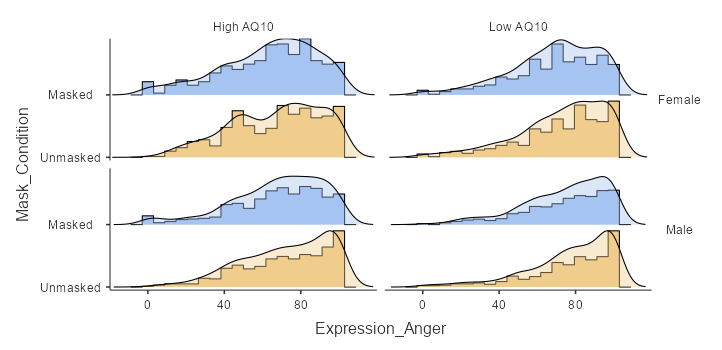


**Confidence_Disgust**


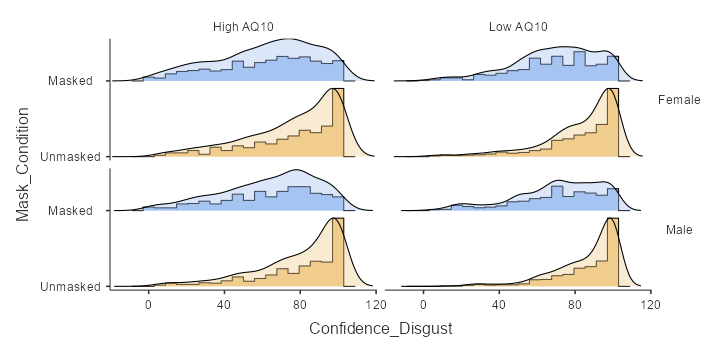


**Expression_Disgust**


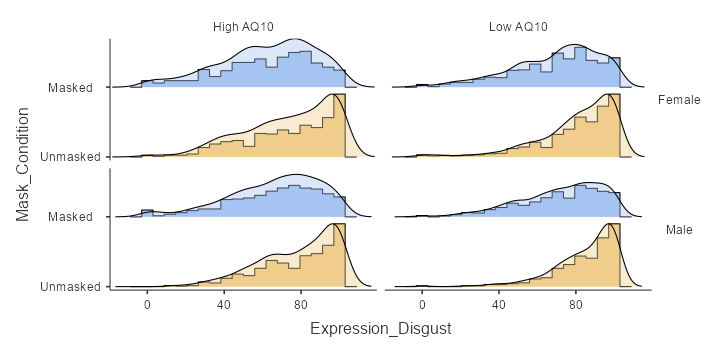


**Confidence_Fear**


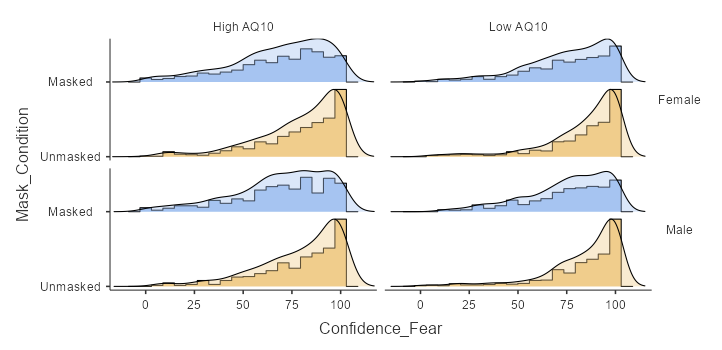


**Expression_Fear**


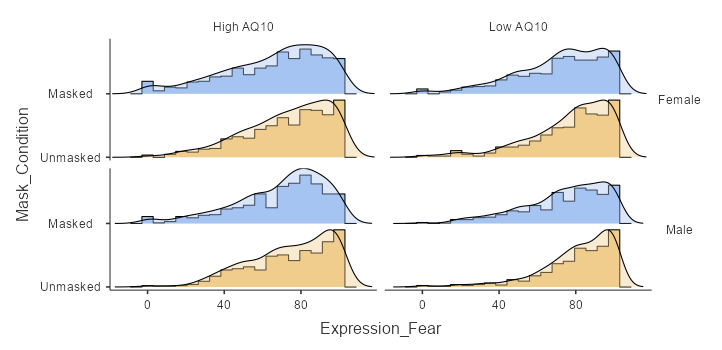


**Confidence_Happiness**


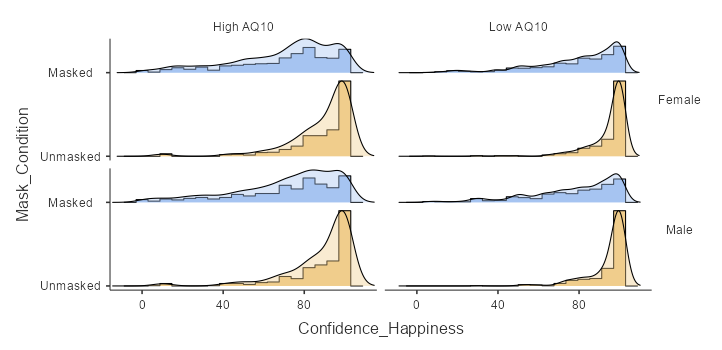


**Expression_Happiness**


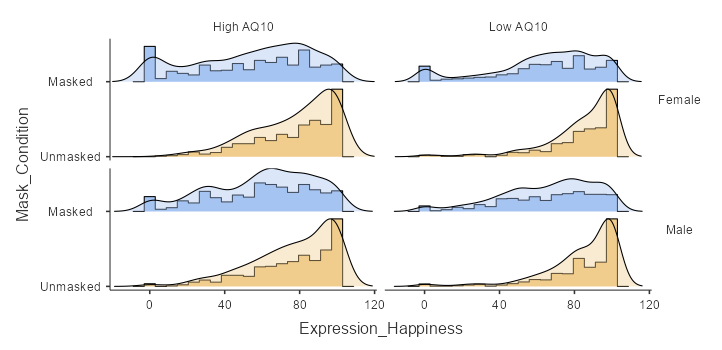


**Confidence_Neutral**


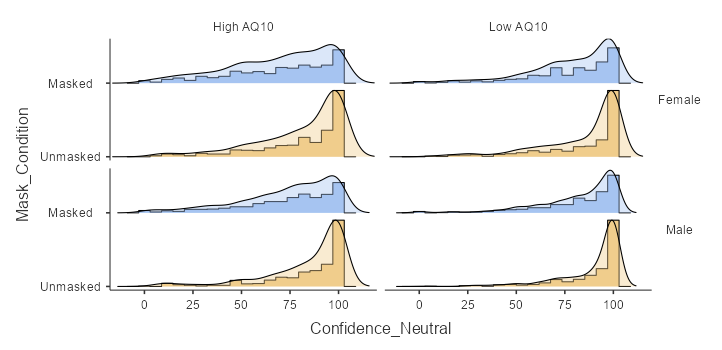


**Confidence_Sadness**


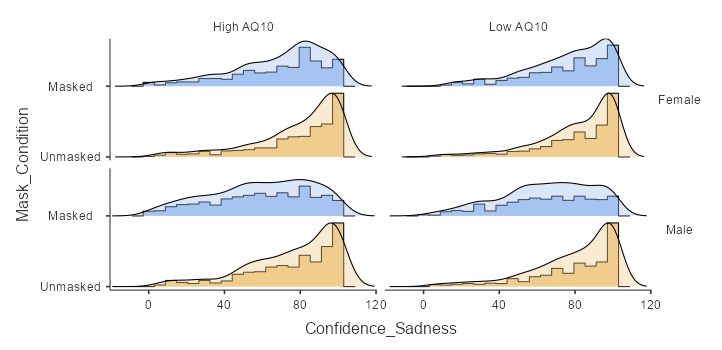


**Expression_Sadness**


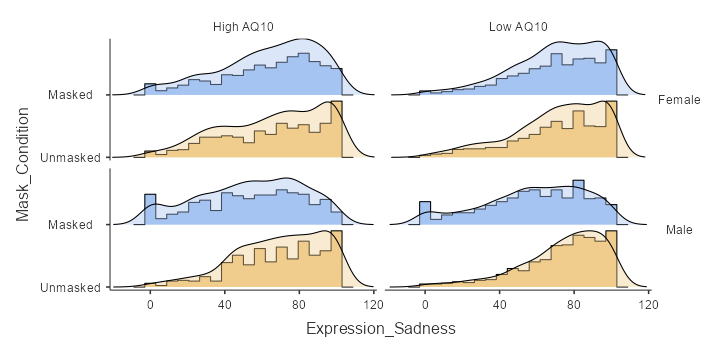

Supplement: S1 File — (DOCX) [file pone.0257740.s001.docx]
